# Deep oncopanel sequencing reveals within block position-dependent quality degradation in FFPE processed samples

**DOI:** 10.1186/s13059-022-02709-8

**Published:** 2022-06-29

**Authors:** Yifan Zhang, Thomas M. Blomquist, Rebecca Kusko, Daniel Stetson, Zhihong Zhang, Lihui Yin, Robert Sebra, Binsheng Gong, Jennifer S. Lococo, Vinay K. Mittal, Natalia Novoradovskaya, Ji-Youn Yeo, Nicole Dominiak, Jennifer Hipp, Amelia Raymond, Fujun Qiu, Hanane Arib, Melissa L. Smith, Jay E. Brock, Daniel H. Farkas, Daniel J. Craig, Erin L. Crawford, Dan Li, Tom Morrison, Nikola Tom, Wenzhong Xiao, Mary Yang, Christopher E. Mason, Todd A. Richmond, Wendell Jones, Donald J. Johann, Leming Shi, Weida Tong, James C. Willey, Joshua Xu

**Affiliations:** 1 Division of Bioinformatics and Biostatistics, National Center for Toxicological Research, U.S. Food and Drug Administration, Jefferson, AR 72079 USA; 2 (Formerly) Department of Pathology, College of Medicine and Life Sciences, The University of Toledo, Toledo, OH 43614 USA; 3 Lucas County Coroner’s Office, 2595 Arlington Ave, Toledo, OH 43614 USA; 4 Immuneering Corporation, 245 Main St, Cambridge, MA 02142 USA; 5 Astrazeneca Pharmaceuticals, 35 Gatehouse Dr, Waltham, MA 02451 USA; 6 Research and Development, Burning Rock Biotech, Shanghai, 201114 China; 7 (Formerly) Pathology and Laboratory Medicine Institute, Cleveland Clinic, 9500 Euclid Avenue, Cleveland, OH 44195 USA; 8 Icahn Institute and Department of Genetics and Genomic Sciences Icahn School of Medicine at Mount Sinai, 1425 Madison Ave, New York, NY 10029 USA; 9 Illumina Inc., 5200 Illumina Way, San Diego, CA 92122 USA; 10 Thermo Fisher Scientific, 110 Miller Ave, Ann Arbor, MI 48104 USA; 11 Agilent Technologies, 11011 N Torrey Pines Rd, La Jolla, CA 92037 USA; 12 Department of Pathology, University of Toledo, 3000 Arlington Ave, Toledo, OH 43614 USA; 13 Department of Pathology, Strata Oncology, Inc., Ann Arbor, MI 48103 USA; 14 Pathology and Laboratory Medicine Institute, Cleveland Clinic, 9500 Euclid Avenue, Cleveland, OH 44195 USA; 15 Department of Medicine, College of Medicine and Life Sciences, The University of Toledo, Toledo, OH 43614 USA; 16 Accugenomics, Inc., 1410 Commonwealth Drive, Suite 105, Wilmington, NC 20403 USA; 17 Center of Molecular Medicine, Central European Institute of Technology, Masaryk University, Kamenice 5, 625 00 Brno, Czech Republic; 18 EATRIS ERIC- European Infrastructure for Translational Medicine, De Boelelaan 1118, 1081 HZ Amsterdam, The Netherlands; 19 Massachusetts General Hospital, Harvard Medical School, Boston, MA 02114 USA; 20 Stanford Genome Technology Center, Stanford University, Palo Alto, CA 94304 USA; 21 Department of Information Science, University of Arkansas at Little Rock, 2801 S. Univ. Ave, Little Rock, AR 72204 USA; 22 Department of Physiology and Biophysics, Weill Cornell Medicine, Cornell University, New York, NY 10065 USA; 23 Market & Application Development Bioinformatics, Roche Sequencing Solutions Inc., 4300 Hacienda Dr, Pleasanton, CA 94588 USA; 24 Q2 Solutions - EA Genomics, 5927 S Miami Blvd, Morrisville, NC 27560 USA; 25 Winthrop P Rockefeller Cancer Institute, University of Arkansas for Medical Sciences, 4301 W Markham St, Little Rock, AR 72205 USA; 26 State Key Laboratory of Genetic Engineering, School of Life Sciences and Shanghai Cancer Hospital/Cancer Institute, Fudan University, Shanghai, 200438 China; 27 Human Phenome Institute, Fudan University, Shanghai, 201203 China; 28 Fudan-Gospel Joint Research Center for Precision Medicine, Fudan University, Shanghai, 200438 China; 29 Departments of Medicine, Pathology, and Cancer Biology, College of Medicine and Life Sciences, University of Toledo Health Sciences Campus, 3000 Arlington Ave, Toledo, OH 43614 USA

**Keywords:** Cancer genomics, Next-generation sequencing, FFPE, Preanalytics, Precision medicine, Oncopanel sequencing

## Abstract

**Background:**

Clinical laboratories routinely use formalin-fixed paraffin-embedded (FFPE) tissue or cell block cytology samples in oncology panel sequencing to identify mutations that can predict patient response to targeted therapy. To understand the technical error due to FFPE processing, a robustly characterized diploid cell line was used to create FFPE samples with four different pre-tissue processing formalin fixation times. A total of 96 FFPE sections were then distributed to different laboratories for targeted sequencing analysis by four oncopanels, and variants resulting from technical error were identified.

**Results:**

Tissue sections that fail more frequently show low cellularity, lower than recommended library preparation DNA input, or target sequencing depth. Importantly, sections from block surfaces are more likely to show FFPE-specific errors, akin to “edge effects” seen in histology, while the inner samples display no quality degradation related to fixation time.

**Conclusions:**

To assure reliable results, we recommend avoiding the block surface portion and restricting mutation detection to genomic regions of high confidence.

**Supplementary Information:**

The online version contains supplementary material available at 10.1186/s13059-022-02709-8.

## Background

Next-generation sequencing (NGS) is now an integral tool in the “precision” cancer care arsenal. Despite excellent performance for somatic mutation calling [1], preanalytical variation continues to limit the quality and quantity of cancer specimens, and this ultimately impacts NGS accuracy and reproducibility [2–4]. One source of preanalytical error stems from formalin fixation and paraffin embedding (FFPE). FFPE processing of tumor specimens is central to histologic diagnosis of cancer and subsequent sub-classification, grading, staging, and adequacy assessment for ancillary studies in routine clinical workup [5]. For this reason, many ancillary prognostic and treatment markers are optimized for FFPE tissue [6, 7]. However, FFPE processing harbors substantial and highly variable effects on nucleic acid quality and quantity [8]. When FFPE effects are combined with a growing trend toward limited specimen size and quality, the potential for error is compounded [9]. Thus, platforms for targeted NGS analysis of FFPE clinical specimens must be subjected to rigorous analytical validation [10–12]. It is of utmost importance that we understand the factors that influence the reproducibility and accuracy of NGS testing in FFPE tissue so that this powerful technology is optimized for implementation in cancer care.

The process of formalin fixation and paraffin embedding includes several steps with varying degrees of (1) fixation, (2) progressive dehydration, (3) clearing, and (4) molten paraffin infiltration [13]. For each step, clinical labs independently establish the ideal amount of time, temperature, pressure, agitation, and reagent composition, typically with the primary goal of optimizing quality for histomorphologic assessment [13, 14]. Some guidelines do exist for specific preanalytical FFPE metrics (e.g., fixation time). For example, the College of American Pathologists recognizes that fixation time can influence accuracy and reproducibility of ancillary tests performed on FFPE such as ER/PgR immunohistochemistry and HER2 in situ hybridization, and for these specimens, formalin fixation should be limited to > 6 or < 72 h of total formalin exposure [6, 7]. Still, false somatic mutation calling rates by NGS vary greatly for FFPE specimens from different, and even within the same, anatomic laboratories [15]. Of even greater concern, FFPE-derived sequencing errors may arise at clinically relevant loci and at actionable allelic frequencies [16]. Working groups are beginning to formulate and extend more detailed recommendations on preanalytical controls [4]. While it is important that these practice recommendations are based on human biospecimen research data, some important questions may benefit from additional studies using carefully contrived sample sets as conducted here.

In this study, the Oncopanel Sequencing Working Group of the FDA-led Sequencing Quality Control Phase II (SEQC2) consortium extended the study [1] to investigate the impact of FFPE processing. This line of inquiry has served to address the insufficiency of well-controlled data regarding FFPE tissue, its preanalytical metrics, and impact on NGS accuracy and reproducibility [17]. Here, we adopted an easy-to-follow FFPE preparation protocol (see the “Materials and methods” section for details) that is highly analogous to clinically obtained cell block cytology sample processing and can be readily applied to existing reference cell line materials [18–20]. The benefits of the approach used here are that (1) reference variant data and the ability to compare with numerous orthologous molecular methods provide robust information for accuracy and reproducibility studies, (2) genetic heterogeneity across replicate measures is minimized (a common challenge in clinical FFPE NGS studies), and perhaps most importantly (3) preanalytical FFPE effects on technical artifacts can be well-documented and controlled, enabling future meta-analysis and subsequent guideline recommendations. In this investigation, cultured cell samples from a diploid cell line [18] (agilent male lymphoblast cell line) were subjected to varying formalin fixation times between 1 and 24 h prior to tissue processing, similar to what is experienced for many specimens in an anatomic pathology laboratory [4, 21, 22]. FFPE sectioning samples at multiple locations within each FFPE block were selected and distributed to four independent laboratories for targeted NGS following amplicon or hybrid capture enrichment. Based on reference variant data, we identified false-positive (FP) calls and estimated FP rate (FPR) within each panel’s targeted region—a key quality control metric demonstrated first in our cross oncopanel investigation [1]. Comprehensive analysis was then conducted on FP calls and FPR to decipher the effects of FFPE factors including formalin fixation time and tissue block section position.

## Results

### Overview of study design and analysis

**Fig. 1:**
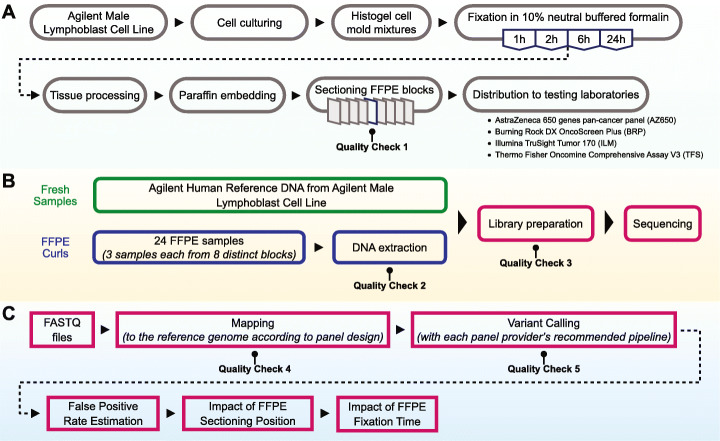
Overview of study design. **A** FFPE sample preparation workflow with four different formalin fixation times: 1, 2, 6, and 24 h. **B** Oncopanel sequencing experiments with in-laboratory DNA extraction. **C** Panel-specific variant calling followed by uniform and integrated analysis to assess the impacts of formalin fixation time and by sample position in the FFPE block

To investigate the effect of formalin fixation time and tissue block section position on targeted NGS analysis of FFPE specimens, we designed a comprehensive study querying several key components. Figure 1 displays the flow of three major components: FFPE sample preparation (Fig. 1A), sequencing experiments with four diverse oncopanels (Fig. 1B), and data quality control and analysis (Fig. 1C). High-quality genomic DNA from a single diploid cell line (agilent male lymphoblast cell line) was sequenced with multiple oncopanels and technical replicates in our companion study [1]. These datasets enabled the establishment of a known variant set for each panel and the subsequent detection of artifacts induced by the FFPE process. Cultured cell populations were used to make FFPE samples with four different formalin fixation time. Equal amounts of cultured cells were mixed in each gel matrix mold, followed by formalin fixation, routine tissue processing, and paraffin embedding (Fig. 1A). Samples were created by sectioning FFPE blocks as described in the “Materials and methods” section. Based on their estimated cell counts and positions in the FFPE blocks, samples were assigned to two categories: surface (either top or bottom of the blocks) or inner FFPE samples (see the “Materials and methods” section for details). Each laboratory extracted and quantified genomic DNA from 24 samples evenly distributed across eight distinct FFPE blocks. NGS sequencing experiments and subsequent bioinformatics processing were conducted following vendor-recommended protocols (Fig. 1B, see the “Materials and methods” section). These FFPE samples were sequenced by four oncopanels (Additional file 5: Table S1): AstraZeneca 650 genes Oncology Research Panel (AZ650), Burning Rock DX OncoScreen Plus (BRP), Illumina TruSight Tumor 170 (ILM), and Thermo Fisher Oncomine Comprehensive Assay v3 (TFS). Variant calling results and QC data were collected and submitted to the Working Group for integrated analysis (Fig. 1C, see the “Materials and methods” section for details).

Five QC check steps were included in our workflow (Fig. 1): (1) cell count estimation, (2) DNA extraction yield, (3) library construction yield, (4) median depth and library complexity computed from mapped reads, and (5) variant histograms by VAF for the detection of contamination. All QC data are provided in Additional file 2. In addition to the standard QC checks during sample processing and sequencing experiments, the variant histogram by VAF was found to be helpful for identifying contaminated samples in this study (Additional file 1: Fig. S1). As we were plotting the VAF histogram for each sample, we surprisingly noticed handful experiments with an unexpected tail on the left side of 100% VAF. Homozygous germline mutations are expected to be observed with a VAF of 100%, with very little or no leftward shift. Taking sample 6_G_7 as an example, the tail was several times wider than the one for sample 1_H_11 (Additional file 1: Fig. S1A-B). The contamination was then confirmed by the observation that a high number of FP calls in each suspected sample were likely germline variants as they can be found in the Single Nucleotide Polymorphism Database [23] (dbSNP) for human or the Exome Aggregation Consortium [24] (ExAC) sequencing data (Additional file 1: Fig. S1C).

In summary, six samples failed in the ILM experiments based on very low target sequencing depth and low library yield, which may be related to the lower than recommended library input. Four samples failed both attempts of library preparation for TFS and were not sequenced. In each case, those failed samples appeared to have been processed together in one batch, potentially failing at the same step between DNA extraction and library construction. Five samples were likely contaminated during the NGS experiments (two in AZ650, two in BRP, and one in ILM), i.e., after FFPE sample preparation. These fifteen (15) samples were excluded from further analysis on FFPE effects as their experimental QC failure was not related to the FFPE sample processing.

### The main constituents of the known variant set were homozygous or heterozygous germline variants

To differentiate FPs from true variants, it was imperative to first build the set of reference (known) variants for each panel. By aggregating the variants called by over 75% of the fresh gDNA samples (16 for ILM or 12 for BRP and TFS) that were free of FFPE damage, we generated a set of known variants for each panel. AZ650 was not included in our companion study of multiple oncopanels using fresh gDNA samples; thus, its known variant set was generated from 21 FFPE samples that each reported a similar number of variants (~ 850). To determine FPs introduced by FFPE processing, variant calls from each FFPE sample were compared with the known variant set for each respective panel.

**Fig. 2:**
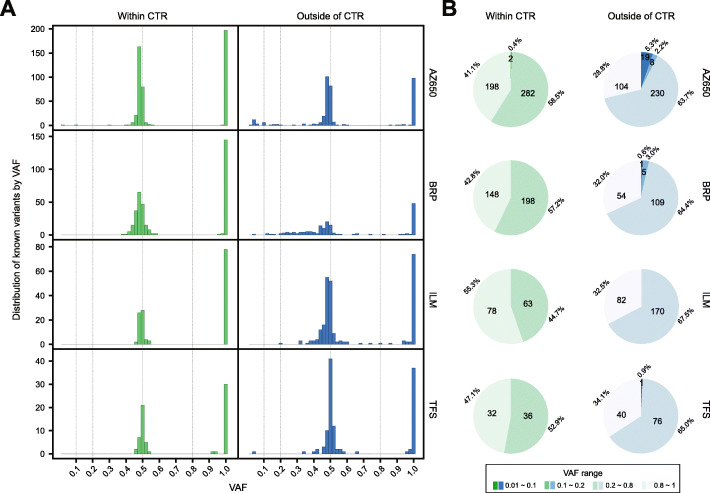
Histogram and pie chart distributions of known variants across VAF ranges confirming that most were homozygous or heterozygous germline variants. **A** Distribution of the count of known variants within (green) and outside (blue) of the consensus high confidence targeted region (CTR). **B** Count and percentage of known variants across four VAF ranges

We expected to detect only homozygous and heterozygous variants in the known variant sets since a diploid cell line was used. In general, the variant allele frequencies (VAFs) were close to either 0.5 or 1 (Fig. 2A). Moreover, the distribution plot revealed few variants falling between VAF 0.1–0.3 and 0.7–0.9 (Fig. 2A). Thus, we choose 0.2 and 0.8 as the boundaries for heterozygous and homozygous variants, respectively. We classified the known variant set into four groups by the VAF value: homozygous variants (VAF > 0.8), heterozygous variants (0.2 < VAF ≤ 0.8), and two additional low VAF ranges separated by a VAF at 0.1. Interestingly, slightly more reference variants fell into the lower VAF ranges for AZ650 due to the lack of fresh gDNA samples for generating the known variant set. Overall, the main constituents of the known variant sets are homozygous and homozygous variants, despite the region or panel differences. The known variants are listed in Additional file 3 for each panel.

Variants located outside of the high confidence consensus targeted region (CTR) displayed greater VAF dispersion than those within the CTR (Fig. 2A; see the “Materials and methods” section for details), particularly for the BRP, ILM, and TFS panels. The number of low VAF variants increased outside of the CTR (Fig. 2B). More specifically, for variants called within the CTR, only two variants (0.4%) were in the two low VAF ranges for AZ650. In contrast, among the variants called by AZ650 outside of the CTR, 27 (> 7%) fell into the low VAF ranges (Fig. 2B). The increased low VAF variants outside of the CTR were also observed in the BRP and TFS panels, though their proportion relative to all variants remained low (Fig. 2B). The lower reliability for calling variants outside of the CTR observed in this study is consistent with the observations in related SEQC2 studies [18]. This led to a much higher FPR outside of the CTR than the corresponding one within the CTR for each sample across all four panels (Additional file 2 and Additional file 1: Fig. S2 ), again reinforcing the findings and recommendation regarding genomic regions from a companion study [1]. To minimalize the impact of region complexity on FP assessment, we confined further analysis on FFPE effects to the CTR.

### Surface FFPE samples showed significantly more FFPE damage and artifacts due to hydrolytic deamination

**Fig. 3:**
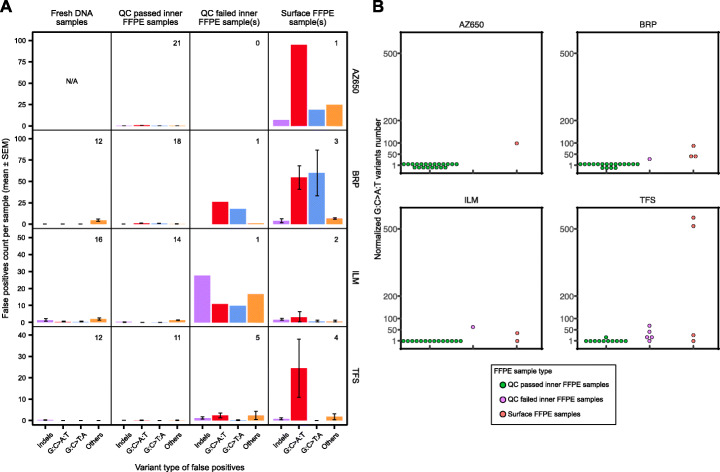
Counts and distribution of false-positive calls by variant types within the consensus targeted region (CTR) indicated more FFPE damage in surface FFPE samples. **A** The average number of false-positive calls is plotted with standard error of the mean (SEM) for four variant types within the CTR for the fresh DNA and various FFPE sample groups. The variant types were (1) indels, (2) hydrolytic deamination introduced artifacts (G:C>A:T transitions), (3) oxidative damage artifacts (G:C>T:A transversions), and (4) other FP calls. The number of samples for each sample group is inserted at the top right corner of each subplot. **B** Normalized G:C>A:T variants number (number of G:C>A:T variants divided by mean G:C>A:T variants in QC passed inner FFPE sample for AZ650, BRP, and TFS; number of G:C>A:T variants divided by mean G:C>A:T variants in QC failed inner FFPE sample for ILM) for each panel over three FFPE sample groups (QC-passed inner FFPE samples vs QC-failed inner FFPE samples vs surface FFPE samples)

In this study, FFPE processing-associated FPs were assessed in relationship to key QC criteria, including measurements of cell count, DNA input, deduplicated sequencing depth, library complexity, and VAF distribution (Additional file 2 and Additional file 1: Fig. S1). By combining the QC assessments with the sample’s sectioning position (Additional file 2 and see the “Materials and methods” section for details), we classified all the FFPE samples into three groups: (1) QC-passed inner FFPE samples, (2) QC-failed inner FFPE samples, and (3) surface FFPE samples. Additional file 2 listed the specific QC failures as notes. After excluding one recurrent FP indel called by TFS (see the “Materials and methods” section for details), all the FPs were also classified into four variant categories: (1) indels, (2) hydrolytic deamination introduced artifacts (G:C>A:T SNVs), (3) oxidative nucleotide damage [25] artifacts (G:C>T:A SNVs), and (4) other FP calls. The average FP count per sample was plotted for each sample category (Fig. 3A).

Overall, compared with fresh and QC-passed inner FFPE samples, both surface- and QC-failed inner FFPE samples produced more FP calls. Except for ILM, the surface FFPE samples consistently made more FP calls per sample than the QC-failed inner FFPE samples (Fig. 3A). Furthermore, across all panels, the surface FFPE samples yielded more hydrolytic deamination artifacts per sample than the QC-failed inner FFPE samples. This clear pattern indicated that the main driver of FP calls in surface FFPE samples was the artifacts introduced by hydrolytic deamination (G:C>A:T). To detail the hydrolytic deamination differences, we normalized the G:C>A:T SNV numbers in each sample as described in the “Materials and methods” section. The normalized value is equal to the number of the samples’ G:C>A:T SNVs divided by the mean G:C>A:T SNVs number of all QC passed inner FFPE samples (for ILM, it was the fresh DNA sample group due to no G:C>A:T SNVs found in any of its QC-passed inner FFPE sample) of the panel. The number of G:C>A:T SNVs called in QC-failed inner and surface FFPE samples was higher than that in QC-passed inner FFPE samples. And for some surface FFPE samples of BRP and TFS, the increases were noticeably higher than those for the QC failed inner FFPE samples (Fig. 3B). To statistically quantify the difference of the hydrolytic deamination artifacts between the sample groups, Welch’s *t*-test was used. Compared to QC-passed inner FFPE samples, significantly more hydrolytic artifacts were observed in both the QC-failed inner FFPE samples (*p*-value = 0.01) and the surface FFPE samples (*p*-value = 0.03). While the test between QC-failed inner FFPE samples and surface FFPE samples was not statistically significant, the *p*-value was relatively low (*p*-value = 0.07) considering the small sample sizes of the two groups. Understandably, the surface FFPE samples may have been exposed to more intensive FFPE processing conditions, leading to more FFPE process-associated uracil lesions [8]. Our results indicate that more DNA damage occurred on the surface portions of the sample during the FFPE processing.

All five surface samples with low cell counts (1600–6200 cells per sample) failed the QC checks. This is understandable as the low cell count would lead to low DNA input amounts for the sequencing experiments. Even after excluding them, the QC passing rate among the surface sample group (3 out of 5) was drastically lower than that of the inner FFPE sample group (64 out of 71). The samples taken from the inner portion of a FFPE block had a significantly higher chance to perform well than samples from the surface portion (*p* = 0.044, Pearson’s chi-squared test with Yates’ continuity correction).

### Inner FFPE samples achieved low FPRs similar to fresh gDNA samples

**Fig. 4:**
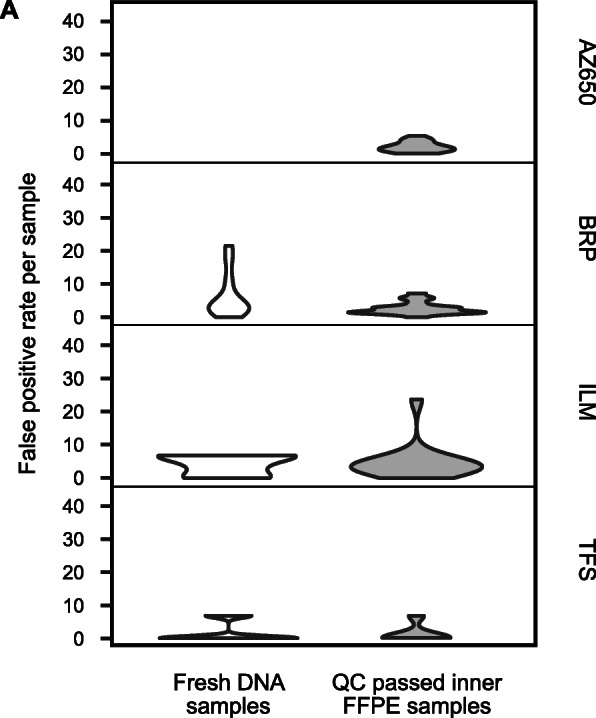
False-positive rates (per million base) of QC-passed inner FFPE samples in comparison with fresh DNA samples. **A** Violin plots of the false-positive rate for fresh DNA samples versus QC-passed inner FFPE samples within the CTR

Our previous statistical tests between three FFPE sample groups demonstrated that the QC-passed inner samples yielded much fewer FP calls than the surface FFPE and QC-failed inner samples. To further assess the impact of FFPE process beyond the surface portions, we evaluated the FPRs of the QC-passed inner FFPE samples compared against fresh samples for panels BRP, ILM, and TFS using a one-tailed *t*-test (Fig. 4A). Within the CTR, there was no statistical difference in FPRs of fresh gDNA and QC-passed inner FFPE samples for any of the three panels: BRP (*p*-value = 0.96), ILM (*p*-value = 0.33), and TFS (*p*-value = 0.65) (Fig. 4A). The QC-passed inner FFPE samples achieved as low FPRs as fresh gDNA samples, i.e., FFPE processing did not lead to any significant increase in the FPR for the inner FFPE samples.

### Formalin fixation time under 24 h showed no impact on FPRs of FFPE samples

**Fig. 5:**
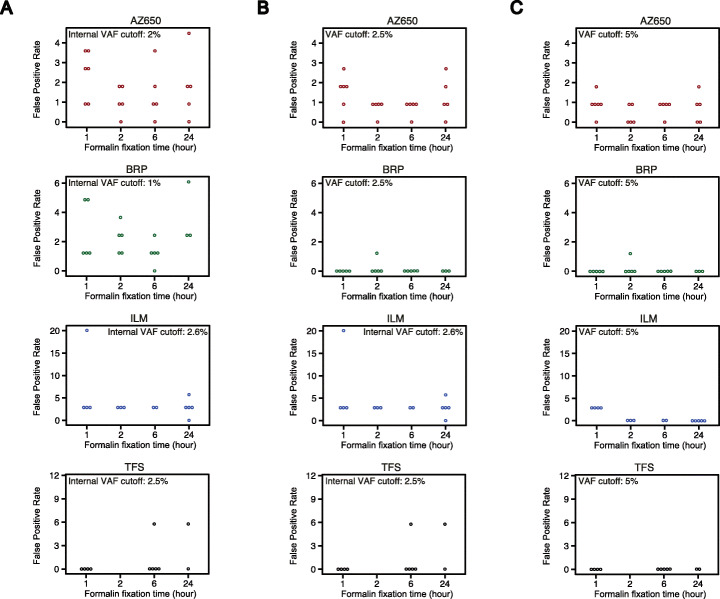
False-positive rates (per million base) of QC-passed inner FFPE samples by 4 different formalin fixation times. **A** For each panel, QC-passed inner FFPE samples are plotted by formalin fixation time (*x*-axis). Each circle represents a sample with its false-positive rate per million bp shown on the *y*-axis. Samples are color coded per panel: red for AZ650, green for BRP, blue for ILM, and black for TFS. Except for TFS, there was no observable effect of formalin fixation time on the quality (measured by false-positive rate) of inner FFPE samples. **B** FPR of QC-passed inner FFPE samples by formalin fixation time (*x*-axis) with an additional 2.5% VAF cutoff. **C** FPR of QC-passed inner FFPE samples by formalin fixation time (*x*-axis) with an additional 5% VAF cutoff

Within the QC-passed inner FFPE samples, we examined whether formalin fixation time would lead to any consistent differences in FPR. This analysis was performed for each panel (Fig. 5A). For each of the four panels, there was no observable effect of formalin fixation time on the FPR of inner FFPE samples. For TFS, the longer fixation group (i.e., 6 h and 24 h) appeared to have two samples of elevated FPRs than the shorter fixation group (i.e., 1 h and 2 h). However, the one-tail *t*-test between these two groups was not significant (*p*-value = 0.086).

In order to optimize the assessment of genomic biomarkers under clinical circumstances such as measurement of tumor mutation burden (TMB), additional VAF filters are necessary to reduce the FPR [1]. By applying a 2.5% VAF cutoff on BRP, nearly all the FPs were removed (Fig. 5A, B), and after applying a 5% VAF cutoff, no more than two FPs were observed in QC-passed inner FFPE samples, and most samples did not report any FPs. This cutoff also further reduced the FPR variation among the sample groups of different formalin fixation time (Fig. 5C). The comparison of FPR indicated that longer formalin fixation time had no detrimental effect on the quality of inner FFPE samples. Taken together, regardless of the fixation time, inner FFPE samples showed no FFPE damage and could achieve the same low FPRs as fresh DNA samples (Figs. 3A and 4A).

## Discussion

The goal of this study was to identify and characterize poorly understood sources of technical variation associated with targeted NGS analysis of variants in FFPE samples. Through the effort of the Oncopanel Sequencing Working Group of the SEQC2 consortium, we used multiple oncopanels and a large set of FFPE samples to systematically survey the effect of formalin fixation time and sectioning position within an FFPE block. Owing to this investigation’s focus on technical variation, a single robustly characterized diploid cell line (agilent male lymphoblast cell line) was used to create FFPE cell blocks with four different formalin fixation times. We adopted an FFPE procedure that is highly analogous to cell block cytology sample processing so that the findings from our study would be relevant to oncopanel sequencing of clinical FFPE samples. We identified multiple previously unrecognized and avoidable sources of variation that, if addressed by appropriate QC measures, should enable more reliable use of FFPE samples.

We leveraged the results from the SEQC2 Oncopanel Sequencing working group that extensively sequenced this cell line in a companion study^1^ to create variant reference sets. However, the variant reference set for AZ650 was generated from FFPE samples that passed stringent QC filters because AZ650 was not included in the companion study of multiple oncopanels with fresh gDNA samples. This might have led to an underestimation of FP rates for samples sequenced by AZ650 because very few variant calls due to FFPE damage may have been included as reference variants. This would not alter our overall conclusion based on the analysis results of FPR because the analysis was carried out for each panel in pairwise comparisons of sample groups to study the effects of formalin fixation time and sample position. Similarly, any limitations or effects of the vendor approved pipelines would not alter our overall conclusion based on pairwise comparisons. It is worthwhile to point out that it is vital to exclude from pairwise comparisons those outliers whose experiment or QC failures were not related to FFPE sample processing.

Given that a QC-related issue in any single step of FFPE processing, sample preparation, library preparation, sequencing, and/or bioinformatics can increase false-positive/negative rates, we sought to survey QC checks throughout the entire process. For the first step (preanalytical QC), cell count and DNA input correlated the FPR to some extents. For the second step (Oncopanel NGS QC), there was a strong correlation between minimum deduplicated sequencing depth (DSD) and FPR. For each hybrid capture panel, we observed some moderate correlation between median DSD and DNA input amount for library preparation. However, there was no correlation between cell count and DNA extraction yield for any panel after excluding those surface samples with very low cell count. The loss of correlation might have been due to high variability in DNA extraction efficiency. For the third step (post-variant call QC), a per-sample VAF histogram demonstrating a long tail from the left side of 100% VAF was likely an indicator of sample contamination. We confirmed the contaminated samples by matching their FP calls among common databases for human genetic variants such as dbSNP and ExAC. This sample contamination check through VAF histogram is possibly applicable to impure tumor samples that may contain DNA from stromal cells. While the exact QC cutoffs employed in this study may not extrapolate perfectly to different panels, tissue samples, and FFPE processes, we expect these QC metrics to remain relevant across oncopanels.

Not all variants fall into equally easy-to-call genomic regions; thus, we queried the impact of variant location on FFPE sample calling. All panels generally showed the lowest FPRs in the CTR (Additional file 2). The FFPE sample processing may interact with the genomic regions and thus further elevate the FPR outside of the CTR. Significant increases were observed in two out of three panels (Additional file 1: Fig. S2). Thus, restriction of variant calls to within the CTR is recommended to boost reliability in the context of FFPE samples. Not surprisingly, a higher VAF cutoff will reduce the FPR. The trade-off was a reduced sensitivity [1], and this may be acceptable in some clinical scenarios if the reduction in sensitivity is moderate. In the case of precious clinical samples from an individual patient, it is highly desirable to make use of (or “rescue”) QC compromised or failed samples through some bioinformatics means. When a higher VAF cutoff is applied to surface samples or QC-failed inner FFPE samples, it resulted in a drastic reduction in sensitivity while the reduction for QC-passed inner samples is moderate and tolerable (Additional file 1: Fig. S3). The higher VAF cutoff was not effective to rescue QC compromised or failed samples in our study. A broad exploration of other bioinformatics methods to remove FFPE-related technical errors without much sacrifice in sensitivity may be interesting for future studies.

Interestingly, physical position within the FFPE block was significantly associated with the level of measured FFPE damage and technical artifacts. Analogous to this, “edge effect” in histologic tissue section staining is observed regularly by pathologists as a source of error in prognostic and treatment marker interpretation [26]. The cause of these surface effects is not completely understood but is thought to stem from a combination of drying artifact, oxidative damage, and formalin fixation damage. In our study, sections taken from the inner portion of the FFPE block were less likely to fail the QC metrics than samples from the surface portions, which often failed QC. This leads to an actionable recommendation that samples from the surface portions of an FFPE block should be avoided if possible when selecting samples for oncopanel sequencing. Our study employed sections of FFPE-prepared cell blocks made from cell culture samples rather than surgically cut tissue samples. For the contrived cytology samples used in this study, the surface portion was usually about 200 μm thick at each end. Further studies are needed to confirm the surface effects and their extent in FFPE-prepared tumor tissue samples. Intact tissue samples harbor widely varying formalin penetration rates, and this can greatly impact formalin fixation time (e.g., brain which is fatty versus muscle with high water content). For clinical samples, it is important to make samples as thin as possible to improve uniformity of fixation in the shortest amount of time possible. A recent publication resulted from the CAP Preanalytics for Precision Medicine Project which recommended that specimen sample thickness be less than 5 mm and total fixation time be between 6 and 24 h for nonfatty tissues to ensure molecular integrity of cancer specimens [4]. Our findings further confirm this recommendation for the formalin fixation time by demonstrating that inner FFPE samples with fixation time up to 24 h can generate reliable oncopanel sequencing data as good as fresh DNA samples.

Taken together, our work advocates for a robust set of QC metrics querying various steps in the process from sample to sequencing to bioinformatics. For the first time, through comprehensive multi-laboratory oncopanel sequencing of 96 samples created under well-controlled FFPE processing, we quantitatively evaluated the effects of formalin fixation time and within-block position on data quality. With a fixation time up to 24 h, there was no observed FFPE effect on the inner samples in terms of the FPR. Regardless of the fixation time, the surface portions showed more FFPE-induced artifacts and higher FPRs and should be avoided when choosing FFPE sections for oncopanel sequencing. To ensure reliable results, our results support the application of strict threshold criteria for cell count, DNA input, allele frequency, and restriction of analysis to genomic regions of high confidence. FFPE samples are archived on a routine basis in pathology departments around the world. By identifying specific quality control factors that affect targeted NGS analysis of FFPE samples, we hope to increase their value in research and clinical diagnostics.

## Conclusions

In this study of NGS artifacts and FPs associated with FFPE, we investigated the effect of formalin fixation time and sectioning position within an FFPE block. With stringent sample quality check and genomic region comparison, and analysis at four laboratory sites, each with a different platform, we found that sectioning position was a key driver of the number of FP calls in FFPE samples. Specifically, surface section FFPE samples were significantly more FFPE-damaged compared to inner FFPE section samples and displayed nucleotide substitutions consistent with hydrolytic deamination. Importantly, the FP rate for inner FFPE section samples was low, similar to fresh DNA samples, and formalin fixation time under 24 h showed no impact on the FP rate for inner FFPE section samples. To assure reliable results, we recommend avoiding the surface portion and restricting mutation detection to genomic regions of high confidence.

## Materials and methods

### Sample preparation, quality check, and distribution

A visual summary was provided as Additional file 4. The Agilent male lymphoblast cell line was cultured in T75 flasks (Corning Catalog No. 10-126-28, Corning Incorporated, Tewksbury, MA) and harvested according to vendor product specifications. The harvested cellular material was combined into a single 15-mL conical tube (Falcon Catalog No. 14-959-53A) and resuspended to a total volume of 1 mL with 10% neutral buffered formalin (NBF) (StatLab Catalog No. 28600, StatLab Medical Products, McKinney, TX). In separate vials, HistoGel specimen processing gel matrix (Thermo Fisher Catalog No. HG-4000-012, Thermo Fisher Scientific, Waltham, MA) was heated to 60 °C for 2 h to liquify and then allowed to cool and equilibrate to 45 °C in a vendor supplied thermal block (Thermo Fisher Catalog No. HGSK-2050-1, Thermo Fisher Scientific, Waltham, MA). Replicate square shape cell block molds were set up (Fisherbrand Catalog No. EDU00553, Thermo Fisher Scientific, Waltham, MA), and into each mold, 500 μL of 45 °C HistoGel was added. Then, 100 μL of NBF-suspended cell line mixture was added. These were immediately and gently stirred to ensure homogeneity of cells within the cooling HistoGel matrix, and then, they were allowed to sit and solidify on the bench top for at least 5 min. Next, for each mold, a micro-spatula was used to carefully dislodge the formed HistoGel embedded cell mixtures, and these were carefully placed into nylon mesh bags (Thermo Fisher Scientific Catalog No. 6774010, Thermo Fisher Scientific, Waltham, MA) to prevent disaggregation during subsequent tissue processing. Each cell mixture block was 2.67 mm thick with a square cross-section of 225 mm^2^. These formed HistoGel cell mixtures in nylon bags were placed into individual tissue processing cassettes (Thermo Fisher Scientific Catalog No. 1000957, Thermo Fisher Scientific, Waltham, MA) and then were submerged in a plastic pail filled with 10% NBF to simulate pre-tissue-processing time-in-formalin delay before batch tissue processing steps.

The sequence described above was performed at 1-, 2-, 6-, and 24-h time points prior to batch tissue processing. All cassettes were then placed into a tissue processor for a “routine” tissue processing run at the University of Toledo Medical Center Department of Pathology (Sakura Tissue Tek VIP 5 Tissue Processor, Saruka Finetech USA, Inc., Torrance, CA). The processor with 14 stations was programmed as follows: (1) 10% NBF for 1 h, (2) 10% NBF for 1 h, (3) 70% ethanol for 1 h, (4) 80% ethanol for 1 h, (5) 95% ethanol for 45 min, (6) 95% ethanol for 45 min, (7) 100% ethanol for 45 min, (8) 100% ethanol for 45 min, (9) xylene for 45 min, (10) xylene for 45 min, (11) paraffin at 60 °C for 30 min, (12) paraffin at 60 °C for 30 min, (13) paraffin at 60 °C for 30 min, and (14) paraffin at 60 °C for 0 min. The processed formalin-fixed paraffin-infiltrated cell blocks were then embedded in paraffin (Sakura Tissue Tek TEC 5 Tissue Embedding Station) to create formalin-fixed paraffin-embedded (FFPE) cell blocks.

Immunohistochemistry of FFPE materials was prepared using Pan Keratin (Ventana Catalog No. 760-2135, Roche Diagnostics International AG, Switzerland) and CONFIRM-anti-CD45 (Ventana Catalog No. 760-2505, Roche Diagnostics International AG, Switzerland) reagents using a Benchmark Ultra Ventana Automated IHC slide staining system. These two IHC stains were used to ensure purity of cell cultures after tissue processing.

Each FFPE cell block was serially sectioned at 5 μm thickness with a microtome. Smaller groups of 8 sections (“curl samples”) were placed into individual low-binding Eppendorf tubes for intra-block comparison of FFPE sampling variation. For cell count and quality control purposes, one alternating section was taken for routine hematoxylin and eosin (H&E) staining. Alternating sets of one H&E slide and 8 sections of FFPE material (“curl sets”) were sectioned until the block was exhausted. The relative position “number” was recorded for each H&E slide and used to mark the corresponding “curl-set” tube. To estimate the cellularity for each tube containing formalin-fixed and paraffin-embedded materials, the average cell count was first computed for the two flanking H&E slides and multiplied by four as a human lymphoblast cell (10–20 μm in size) would likely appear in two adjacent sections of 5 μm thickness. Most cell count estimates ranged from approximately 10,000 to 40,000.

Samples with obviously low counts at the top end of each block were excluded prior to the first recording of relative position. Based on the relative positions and cell count estimates, samples were grouped into three categories: surface (top or bottom) samples with cell count estimates below 50% of the average count per sample, the adjacent surface samples with similar cell counts, and the inner samples. Most inner samples showed cell counts above 20,000. Three adjacent samples were assigned to a second surface category at each end of an FFPE cell block. Occasionally, one or two more adjacent samples were assigned to the second surface category if their cell counts were much closer to the surface samples than the inner samples. Samples of the first surface category usually showed cell counts below 10,000. Cell counts for all available samples and their categories are provided in Additional file 2. All samples were coded by a concatenated string of three fields separated by an underscore “_”: up to two digits for formalin fixation time in hours, a letter in uppercase for FFPE block (“G” or “H”), and up to two digits for FFPE block position. For each formalin fixation time, two FFPE blocks were used, and three samples were taken evenly from each block. A set of 24 FFPE curl samples was then distributed to each testing laboratory.

### Cross-panel targeted NGS testing of FFPE samples

Four laboratories participated in this study and each tested one distinct oncopanel with support from the panel provider. These four panels were AstraZeneca 650 genes Oncology Research Panel (AZ650), Burning Rock DX OncoScreen Plus (BRP), Illumina TruSight Tumor 170 (ILM), and Thermo Fisher Oncomine Comprehensive Assay v3 (TFS). Each laboratory extracted and quantified genomic DNA from the FFPE sections. NGS sequencing experiments were conducted following vendor-recommended protocols, with QC data collected as well. Sequencing data was then processed by the respective oncopanel vendor-recommended bioinformatics pipeline. Detailed information regarding targeted NGS experiment and variant calling method is provided below for each panel. Variant calling results and QC data were collected and submitted to the Working Group for integrated analysis.

### AZ650 panel NGS testing and variant calling

The AZ650 assay is a hybrid capture panel designed by AstraZeneca to perform next-generation sequencing on solid tumors for exploratory evaluation of pan-cancer biomarkers. AZ650 was designed with reference sequences from human genome HG38. DNA extraction was performed on FFPE tissue using the Omega M6958 Kit (Omega Bio-tek, Inc., Norcross, GA) performed on the KingFisher Flex instrument (Thermo Fisher Scientific, Waltham, MA). Extracted DNA was quantitated using the Qubit dsDNA High Sensitivity Kit (cat # Q32854, Thermo Fisher Scientific, Waltham, MA). Each sample was quantitated in duplicate 2 μL reactions, and the average was calculated as the final DNA concentration (ng/μL).

DNA whole-genome libraries were constructed using the Kapa Biosystems HyperPlus kit (cat # 07962428001, Roche Diagnostics International AG, Switzerland) onboard the Beckman Coulter Biomek FxP liquid handling platform (Beckman Coulter, Inc., Brea, CA) with an integrated on-deck Biometra TRobot thermal cycler (Analytik Jena US, Upland, CA). A DNA aliquot was normalized for each sample in 10 mM TRIS-HCl buffer. Enzymatic fragmentation was performed to shear DNA prior to adapter ligation. Unique dual-indexed adapters containing a 6-bp UMI sequence were ligated to the fragmented DNA. The DNA whole-genome libraries were quantitated using the Agilent TapeStation D1000 (cat # 5067-5582, Agilent Technologies Inc., Santa Clara, CA). Quantitation values and fragment lengths sourced from the TapeStation D1000 were used for quality control prior to hybridization capture reaction.

Hybridization capture was performed to enrich for the regions of the genome that comprise the targeted panel. Prior to hybrid capture, whole-genome libraries were multiplexed together in equimolar ratios, and concentrated using a SPRI bead method. The hybridization capture protocol was performed manually using probes produced by IDT and the Roche NimbleGen SeqCap Hybridization and Wash Kit (Roche Diagnostics International AG, Switzerland). Hybrid capture libraries were quantitated using both the Agilent TapeStation D1000 ScreenTape (Agilent Technologies Inc., Santa Clara, CA) and the Kapa Biosystems Library Quantification kit (qPCR, Roche Diagnostics International AG, Switzerland).

Sequencing of each hybrid capture pool was performed on either the Illumina HiSeq 4000 or NovaSeq 6000 sequencers (Illumina, Inc., San Diego, CA). Each pool was normalized to 1 nM and quantitated via TapeStation D1000, then diluted to a final concentration of 200 pM prior to flowcell loading. Sequencing Analysis Viewer (SAV) and the MultiQC tools [27] were used to review the quality metrics generated from the sequencer. Sequencing data was demultiplexed, passed through a bcl-to-fastq conversion program [28] (bcl2fastq v2.20.0.422). FASTQ files were analyzed using pipeline software bcbio-nextgen [29]. Reads were aligned to the hg38 reference using bwa mem v0.7.17 [30], and sequencing duplicates for each UMI were collapsed into a single consensus read using fgbio [31] v1.0.0. Variant calling was performed using VarDict v1.7.0 [32], down to a variant allele frequency (VAF) of 1% (before filtering and curation) and variant effects annotated by snpEff v4.3.1t [33]. All software was run using best practice parameters established within the bcbio workflow or in-house. Mapped UMI consensus reads (in BAM files) and variant calling results (in VCF files) were then provided to the working group for further analysis. The following variant filters were recommended by the panel provider to minimize false-positive calls: (1) a total depth threshold of 100; (2) at least four forward, four reverse, and ten total support reads for the alternative allele; (3) VAF threshold of 2%; and (4) mean position in support reads (pMEAN) [32] greater than 15.

### BRP panel NGS testing and variant calling

The DNA from 24 individual formalin-fixed cell pellets in paraffin scrolls were extracted using the AllPrep DNA/RNA FFPE kit (Qiagen, LLC., Germantown, MD) following the manufacturer’s genomic DNA purification protocol. The extraction process involved deparaffinization, protease digestion, DNA-containing pellet separation, second protease digestion, de-crosslinking, column binding, washing, and elution. After purification, the DNA concentration was quantified using a Qubit Fluorometer with dsDNA HS assay kit (Life Technologies, Carlsbad, CA). The library prep and enrichment process were performed using a Burning Rock HS library preparation kit. The procedure was described previously [1]. In brief, DNA shearing was performed on each FFPE DNA samples using a Covaris M220 for 240s (Covaris Inc., Woburn, MA), with peak incident power = 50 W, duty factor 20%, cycle per burst 200, at 2–8 °C, followed by end repair, adaptor ligation, and PCR enrichment. Approximately 750 ng of purified pre-enrichment library was hybridized to the OncoScreenPlus panel and further enriched following the manufacturer’s instructions. The OncoScreenPlus panel is approximately 1.7M bp in size and covers 520 human cancer-related genes. Final DNA libraries were quantified using a Qubit Fluorometer with dsDNA HS assay kit (Life Technologies, Carlsbad, CA). Agilent 4200 TapeStation D1000 Screen Tape was then performed to assess the quality and size distribution of the library. The libraries were sequenced on a NovaSeq 6000 instrument (Illumina, Inc., San Diego, CA) with 2 × 150 bp pair-end reads with a unique dual index.

After demultiplexing using bcl2fastq v2.20 [28] (Illumina), sequence data were filtered using the Trimmomatic 0.36 [34] with parameters “TRAILING:20 SLIDINGWINDOW:30:25 MINLEN:50.” Sequence data in FASTQ format were mapped to the human genome (hg19) using BWA aligner 0.7.10 [30]. Local alignment optimization, variant calling, and annotation were performed using GATK v3.2.2 [35] with parameters “--interval_padding 100 -known 1000G_phase1.indels.b37.vcf -known Mills_and_1000G_gold_standard.indels.b37.vcf” and VarScan v2.4.3 [36] with parameters “-min-coverage 50 --min-var-freq 0.005 --min-reads2 5 --output-vcf 1 --strand-filter 0 --variants 1 --p-value 0.2.” For SNV and small indels, variants were further filtered using an in-house variant filter pipeline. For each valid variant, the covered raw depth was required to be greater or equal to 50 (DP ≥ 50) and at least 5 mutation supporting count (AD ≥ 5); minor allele frequency was required to be greater than 0.01 (AF ≥ 0.01). In order to further filter out false positives, only variants with at least 6 unique fragments support or 2 unique paired fragment support, i.e., within overlapping region between read pairs, were kept. After filtering, remaining valid variants were annotated with ANNOVAR 20160201 [37] and SnpEff v3.6 [33].

### ILM panel NGS testing and variant calling

FFPE curls were de-paraffinized with xylene followed by an ethanol wash. Briefly, 1 mL xylene was added to each tube with the FFPE sample, vortexed vigorously for 10 s, and centrifuged at 20,000×*g* for 2 min. The supernatant was carefully removed. Then, 1 mL 96–100% ethanol was added to the pellet, mixed by vortexing, and centrifuged at 20,000×*g* for 2 min. Ethanol was removed, and the pellet was air-dried. DNA extraction from the pellet was performed using the QIAGEN Allprep DNA/RNA FFPE kit (Qiagen, LLC., Germantown, MD) on a QIAcube. DNA concentrations were determined by fluorometric quantitation using a Qubit 2.0 Fluorimeter with a Qubit DNA dsDNA HS Assay Kit (Thermo Fisher Scientific, Waltham, MA).

Library preparation was carried out using the TruSight Tumor 170 Assay (Illumina, Inc., San Diego, CA) following the manufacturer’s instructions, except with a lower amount of DNA. Briefly, 30 ng DNA from each sample, except 24G15, 24H10, and 24H15 with 25, 21, and 8 ng DNA, respectively, was fragmented on a Covaris Ultrasonicator (Covaris Inc., Woburn, MA) using the following setting: peak incident power 50 W, duty factor 30%, cycles per burst 1000, treatment time 270 s, and temperature 20 °C. The fragmented DNA was processed through end repair, A-tailing, adapter ligation, and index PCR, enriched by the hybridization-capture method, amplified by final PCR, normalized by bead-based normalization, and pooled for sequencing. Twenty-four samples were batched for each library prep, and libraries from every 8 samples were pooled and sequenced on the Illumina NextSeq 550 instrument using the NextSeq High Output Kit (Illumina, Inc., San Diego, CA). BaseSpace Sequence Hub was used to set up the sequencing run, perform the initial quality control, and generate FASTQ files for each sample.

Variant calling was performed in BaseSpace Sequence Hub. Briefly, high-level sequencing run metrics were evaluated to generate a Run QC Metrics report. Next reads were converted into the FASTQ format using bcl2fastq [28]; adapters were trimmed and then reads were aligned to the human genome version hg19 using the iSAAC aligner [38]. Indel realignment was performed and then candidate variants were identified using the Pisces variant caller [39], with a fixed lower limit cutoff for variant allele fraction of at least 2.6%. Variant calls were further compared against a baseline of normal samples to remove systematic false positives.

### TFS panel NGS testing and variant calling

Genomic DNA was extracted from 24 samples of sectioned material using the ALLPrep DNA/RNA FFPE kit (cat # 80234, Qiagen, LLC., Germantown, MD). Samples were eluted in 15 μL from which 1 μL of material was used for quantification. Extracted material was prepared for quantification with a Qubit™ dsDNA HS (High Sensitivity) Assay Kit using 1 μL of sample material in 200 μL of Qubit solution (Thermo Fisher Scientific, Waltham, MA). Concentration readings in ng/μL from the Qubit Fluorometer were used to calculate 20 ng DNA input in a maximum volume of 7.5 μL into the library preparation.

Libraries were generated using the Ion AmpliSeq Oncomine Comprehensive panel versions 3.0 from Thermo Fisher Scientific (Waltham, MA) as described [40], and 17 amplification cycles were performed as suggested for FFPE samples. Final library quantification was performed using real-time PCR (QuantStudio), and the values were given by the instrument in pM. Fifteen out of 24 libraries passed the QC threshold of 50 pM and were successfully sequenced. Five out of 24 libraries did not pass the QC threshold of 50 pM (libraries < 50 pM). These samples were cleaned using 1.8 X Ampure beads, eluted in 15 μL then Qubit quantified using 1 μL from the cleaned material. The input DNA amount to be used into library preparation was determined using the samples’ post clean-up Qubit values multiplied by 7.5, which is the maximum volume of input material for library preparation. The newly constructed libraries passed the QC threshold of 50 pM and were successfully sequenced.

The remaining 4 out of 24 libraries did not pass the QC threshold of 50 pM (libraries < 50 pM). These samples were cleaned using 1.8 X Ampure beads, eluted in 15 μL then Qubit quantified using 1 μL from the cleaned material. Qubit values were too low to be detected, so 7.5 μL was used as input for library preparation. The newly constructed libraries did not pass the QC threshold of 50 pM and therefore were not sequenced (libraries < 50 pM).

All barcoded samples were sequenced on the Ion S5 XL System (Thermo Fisher Scientific, Waltham, MA). Analyses of sequencing raw data were performed with the Ion Torrent Suite (5.10.0). After base calling, reads were aligned with the TMAP module, and variant calling was performed with Torrent Variant Caller (TVC), a variant calling module optimized for Ion Torrent data. The default thresholds used for SNVs and indels were 2.5%. Finally, a series of post-calling filters were applied to variant calls to eliminate potential artifacts by fitting the statistical model of flow signals for the observed reference and non-reference alleles. For more details, please see the description of its bioinformatics pipeline in the cross-laboratory oncopanel study [1] where this panel was used to test a set of reference samples including Agilent male DNA control.

### Identification and analysis of false-positive calls

#### Consensus high confidence targeted region (CTR)

In our collective consortium effort to establish a verified genomic reference material [18] suitable for assessing the performance of oncology panels in detecting small variants of low allele frequency, a consensus high confidence region for targeted NGS was adopted and later tested in a cross-laboratory oncopanel study [1] of eight oncology panels where the CTR was shown to reduce the rate of false-positive variant calls. The CTR resulted from intersecting exonic coding regions, the NIST high confidence region, and the targeted regions of four whole-exome sequencing panels. Low-complexity regions were then removed from the CTR.

#### VCF file cleaning procedures

VCF files provided by each panel vendor were cleaned through a series of procedures. First, each original VCF file was converted into standardized VCF format. INDELs were then normalized with left alignment and trimming using GATK [35]. Complex variants were decomposed with RTG “vcfdecompose.” After that, only variants within the panel regions (excluding the low complexity regions) were kept. Specifically, for VCF files from the TFS panel, we also removed the blocklist variants provided by Thermo Fisher Scientific. To further remove the less confident variants, VAF thresholds recommended by the panel providers were applied for each panel: 2% for AZ650, 1% for BRP, 2.6% for ILM, and 2.5% for TFS. All downstream analyses were applied to the cleaned VCF files.

#### Generation of known variant set for each panel

The known variant set was generated from the fresh DNA samples of each panel. Any variants called in over 75% of all samples were each considered as a known variant. However, due to only three fresh DNA samples that were sequenced for AZ650 panel, instead of using the fresh DNA samples, we adopted high-quality FFPE samples with deep sequencing depth (after excluding two contaminated samples) for generation of the known variant set. For the ILM panel, we excluded 6 fresh DNA samples for their low median depth (less than 850). Finally, 20 FFPE samples from AZ650, 12 fresh DNA samples from BRP, 10 fresh DNA samples from ILM, and 12 fresh DNA samples from TFS were used for the known variant set generation for each panel.

#### The false-positive estimation and type classification

After the cleaning procedures, any variants called by a panel that were not in the known variant set for that panel were determined as false-positive calls. Two metrics were used in the study. The false-positive call count was the number of false-positive variants for each library, and the false-positive rate was then estimated as the ratio of false-positive calls out of every million positions defined by each panel. Besides the false-positive estimation from the originally cleaned library, false positives could be further reduced by applying more stringent cutoffs. Two types of additional filters were adopted in this study, VAF cutoffs from the panel default VAF threshold to 10% and alternative allele depth cutoffs from 0 to 30. For analysis purposes, besides evaluating the impact of various factors on the FP rate, we also examined the effect per variant type by grouping variant calls into four types: INDELs, G:C to A:T transitions, G:C to T:A transversions, and any other variants.

#### Recurrent false-positive indel filter

During the false-positive estimation, we noticed one specific false-positive indel called by multiple FFPE samples processed with the TFS panel. Further analysis indicated this indel FP call was panel dependent, as no other recurrent FP indels were found in other panels, and thus it could be caused by Ion Torrent technology. To remove this bias that not related with the FFPE damage, we applied an additional recurrent false-positive indel filter for panel, and only that one indel from TFS that we firstly noticed was removed eventually.

## Supplementary Information

**Additional file 1: Fig. S1.** Detection and confirmation of sample contamination. **Fig. S2.** Violin plots of the false positive rate for fresh DNA samples versus QC passed inner FFPE samples in three panel regions (whole panel, within the CTR, or outside of the CTR). **Fig. S3.** Impact of additional VAF cutoffs on the FPR and sensitivity for each FFPE sample type.

**Additional file 2.** A multiple tab Microsoft Excel file of FFPE experiment information, extensive quality check data and analysis results for FFPE samples, and analysis results for free DNA samples.

**Additional file 3.** List of known variants for four participating oncology panels.

**Additional file 4.** Visual Summary for FFPE materials preparation.

**Additional file 5: Table S1.** List of detailed information for four participating oncology panels.

## Data Availability

NGS sequencing data (in FASTQ or BAM format) from 92 samples have been deposited in the National Center for Biotechnology Information (NCBI) BioProject repository under the project PRJNA730887 [41]. Variant call results (in VCF format) and panel BED files have been made publicly available through FigShare [42].
