# Supplementary material for: Deep oncopanel sequencing reveals within block position-dependent quality degradation in FFPE processed samples
Source: Genome Biol. 2022 Jun 29;23:141. doi: 10.1186/s13059-022-02709-8 (PMC9241261; doi:10.1186/s13059-022-02709-8)
Supplement: Supplementary file 1 — Additional file 1: Fig. S1. Detection and confirmation of sample contamination. Fig. S2. Violin plots of the false positive rate for fresh DNA samples versus QC passed inner FFPE samples in three panel regions (whole panel, within the CTR, or outside of the CTR). Fig. S3. Impact of additional VAF cutoffs on the FPR and sensitivity for each FFPE sample type. [file 13059_2022_2709_MOESM1_ESM.pdf]

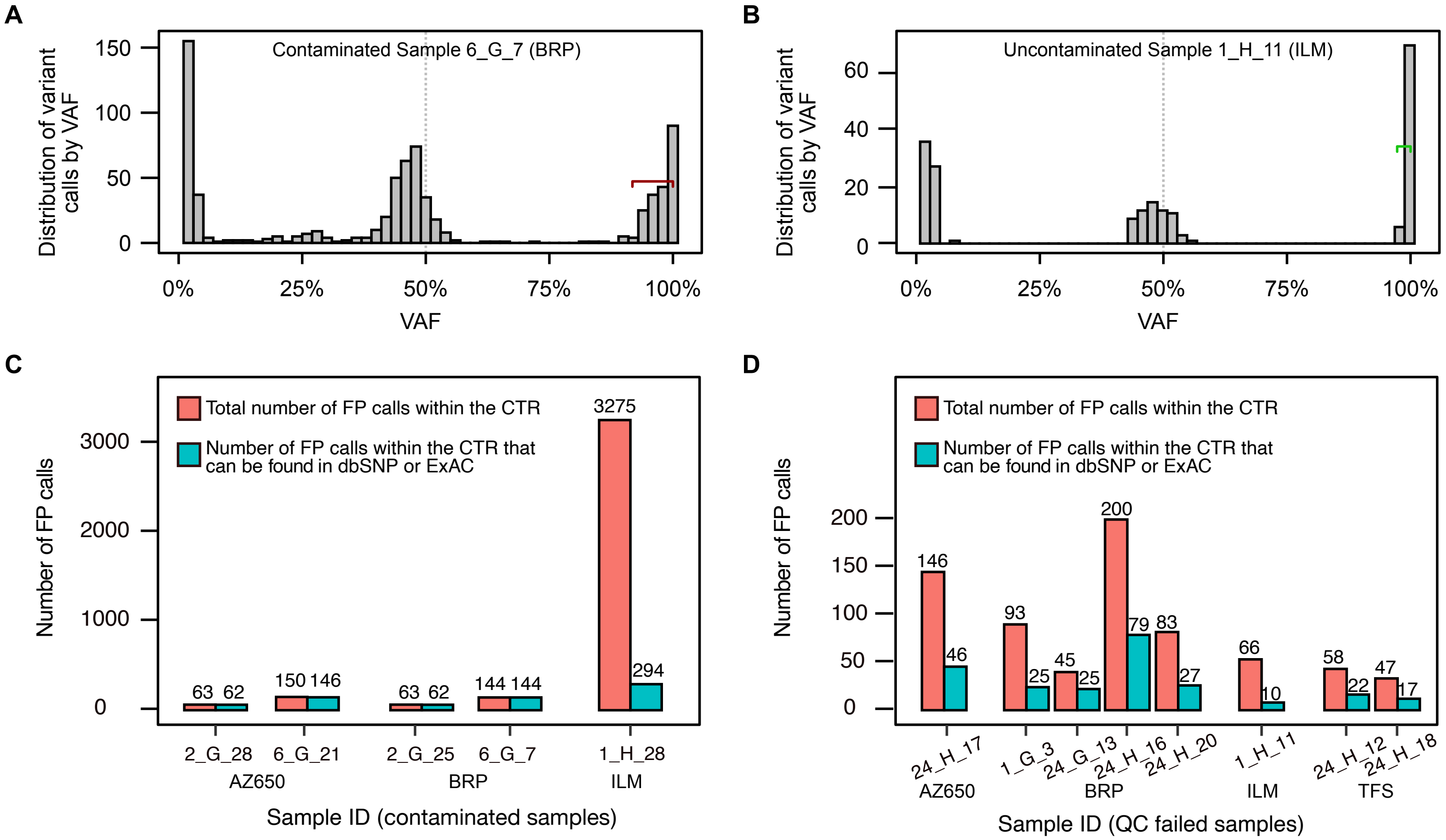

Fig. S1: **Detection and confirmation of sample contamination.** (A) In this VAF histogram, a long tail from the left side of 100% VAF was observed in sample 6_G_7 for homozygous variants, which signals potential contamination. Similar spreads in homozygous variants VAF tail were observed in the histograms of four other samples (not shown). (B) As a comparison, the VAF histogram of sample 1_H_11 was plotted. The tail for homozygous variants was small. (C) A bar chart for 5 contaminated samples was plotted for the number of FP calls within the CTR and the subset of them that can be found in the Single Nucleotide Polymorphism Database (dbSNP) for human or the Exome Aggregation Consortium (ExAC) sequencing data. A high number of FP calls in each suspected sample were likely germline variants as they can be found in these two common databases for human genetic variants. Contaminated samples from the same FFPE block also displayed a low level of overlapping among the FPs. Samples 2_G_25 and 2_G_28 were both sectioned from the same FFPE block 2_G. They were tested by BRP and AZ650, respectively. Within the CTR targeted by both panels, 60 and 43 FPs were reported respectively with only 27 being shared. Likewise, 103 and 135 FPs were reported in samples 6_G_7 and 6_G_21, respectively, with only 51 being shared. Thus, these samples were likely contaminated separately by different human samples. The extremely high number (i.e., 1047, see Supplementary File 1) of indels reported in sample 1_H_28 within the CTR, where no indel was confirmed by the ILM panel for the cell line (see Supplementary File 2), pointed to a contamination by non-human sample(s). The random positions of contaminated samples within the FFPE blocks and the observed inconsistency of contamination between each pair of FFPE blocks (i.e., observations of sample contamination in blocks 1_H, 2_G, and 6_G but none in blocks 1_G, 2_H, and 6_H) undergoing the same duration (i.e., 1, 2, and 6 hours respectively) of formalin fixation and subsequent tissue processing side by side further supported the conclusion that the few contamination events occurred separately after the FFPE sample sectioning step and likely during the sequencing experiments. (D) Similar analysis on FP calls was plotted for eight QC failed FFPE samples with considerable FP calls. Each of their VAF histogram plots (not shown) produced a narrow tail for homozygous variants in the respective panel's targeted region. In contrast to the contaminated samples where the FPs were dominated by SNPs, on average only 34% (16% - 56%) of FPs in these QC failed samples were SNPs in the dbSNP database or the ExAC dataset. This confirmed that these QC failed samples (24_H_17, 1_G_3, 24_G_13, 24_H_16, 24_H_20, 1_H_11, 24_H_12, and 24_H_18) were free of contamination. Their QC failure may be related to FFPE processing and were thus included in the further analysis on FFPE effects.

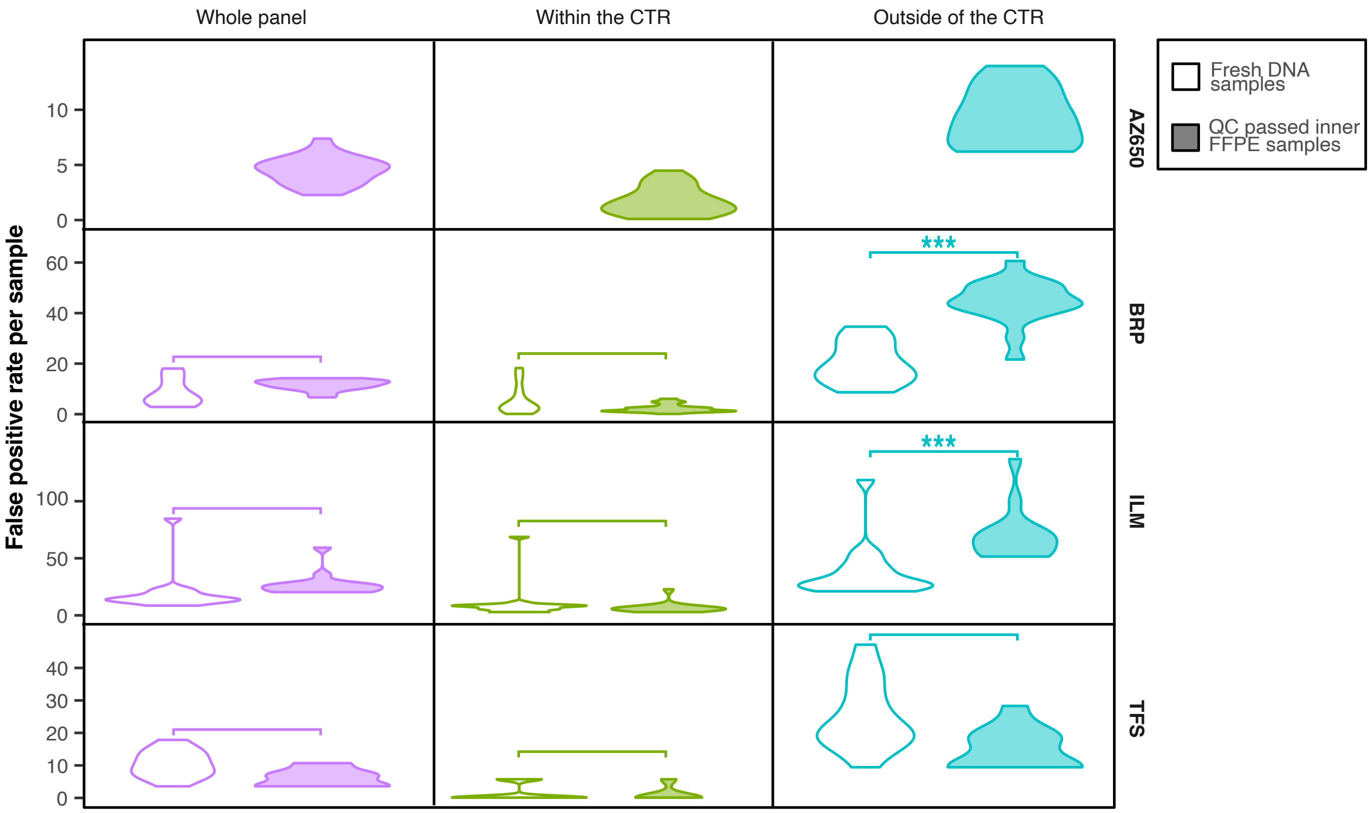

Fig. S2: **Violin plots of the false positive rate for fresh DNA samples versus QC passed inner FFPE samples in three panel regions (whole panel, within the CTR, or outside of the CTR).** The asterisk symbols represent the significance level of the true difference in means is less than 0 ($*$: p< 0.05, $**$: p< 0.01, $***$: p< 0.001). Similar false positive rates (per million base) were achieved by QC passed inner FFPE samples in comparison to fresh DNA samples within the CTR. Significantly higher false positives rates were observed outside of the CTR than the corresponding ones within the CTR across panels and sample types. Furthermore, the false positive rates outside of the CTR in QC passed inner FFPE samples were significantly higher than those from fresh DNA samples for both BRP (p-value = 2.83 * 10^{-8}) and ILM (p-value = 4.13 * 10^{-4}) panels.

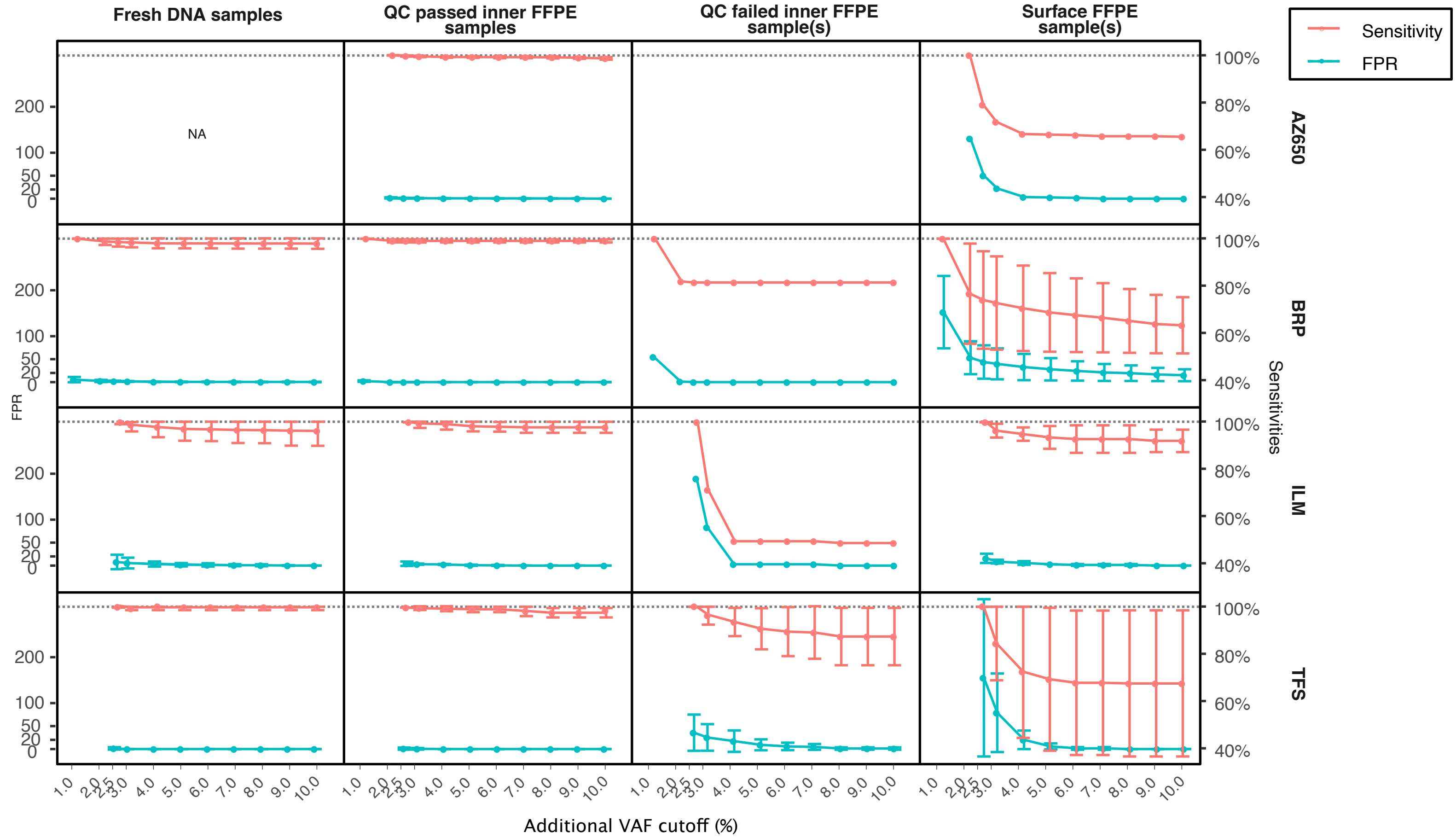

Fig. S3: **Impact of additional VAF cutoffs on the FPR and sensitivity for each FFPE sample type.** Here we show the mean FPRs and mean sensitivities under additional VAF cutoffs from 1% to 10%, with error bars indicate ± standard deviations. As the variants with higher VAF were unlikely to be removed through the additional VAF cutoffs below 10%, we calculated the sensitivities using only the known variants with the expected VAF below or equal 80%.
