# Supplementary material for: Deep oncopanel sequencing reveals within block position-dependent quality degradation in FFPE processed samples
Source: Genome Biol. 2022 Jun 29;23:141. doi: 10.1186/s13059-022-02709-8 (PMC9241261; doi:10.1186/s13059-022-02709-8)
Supplement: Supplementary file 4 — Additional file 4. Visual Summary for FFPE materials preparation. [file 13059_2022_2709_MOESM4_ESM.pdf]

Visual Guide to FFPE materials preparation

Lucas County Coroner's Office, Toledo, Ohio
tblomquist@lcco.co.lucas.oh.us

University of Toledo Medical Center – Pathology
thomas.blomquist@utoledo.edu

Culture of Agilent B-Lymphoblast Male cell line

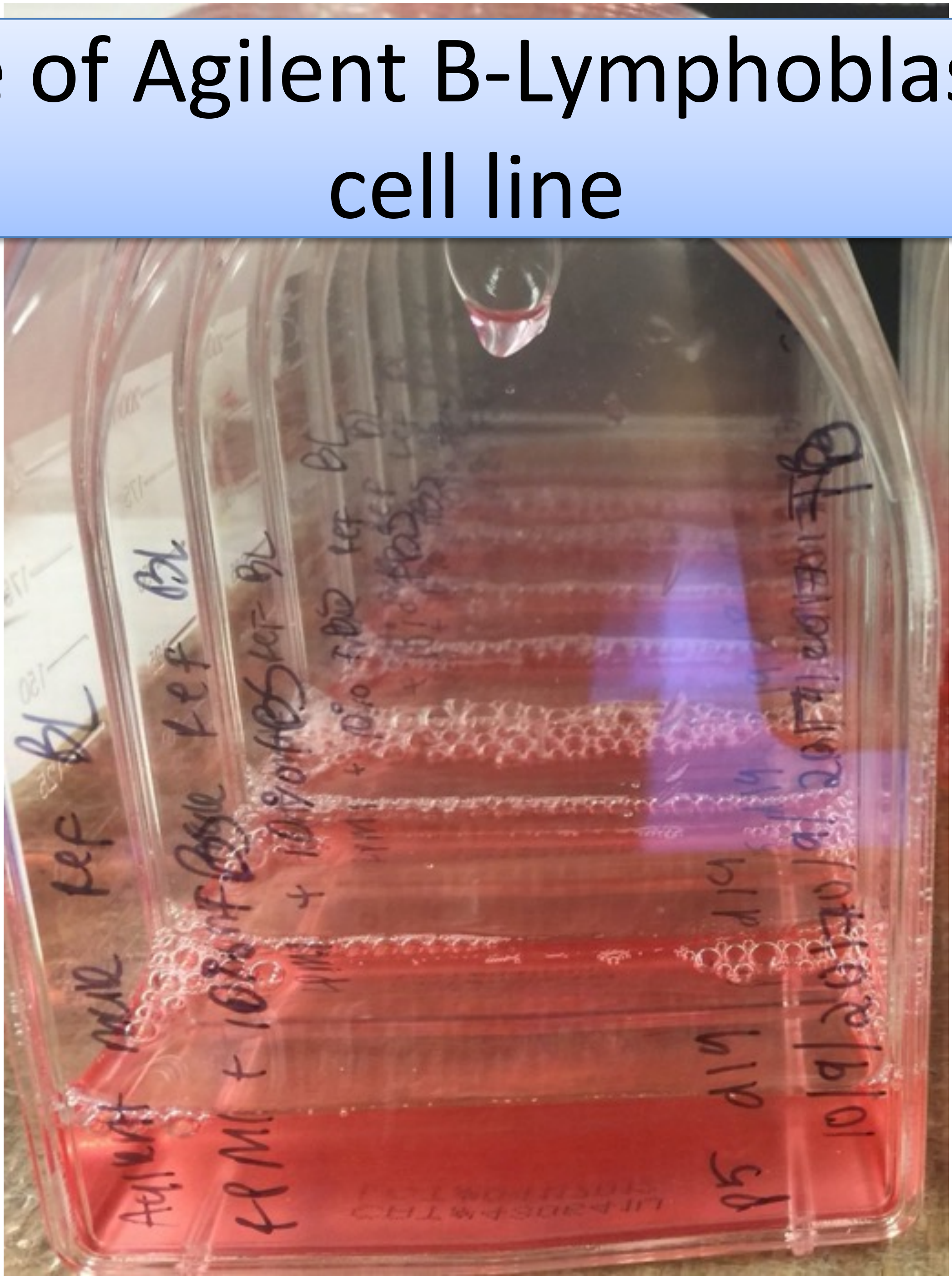

Harvesting

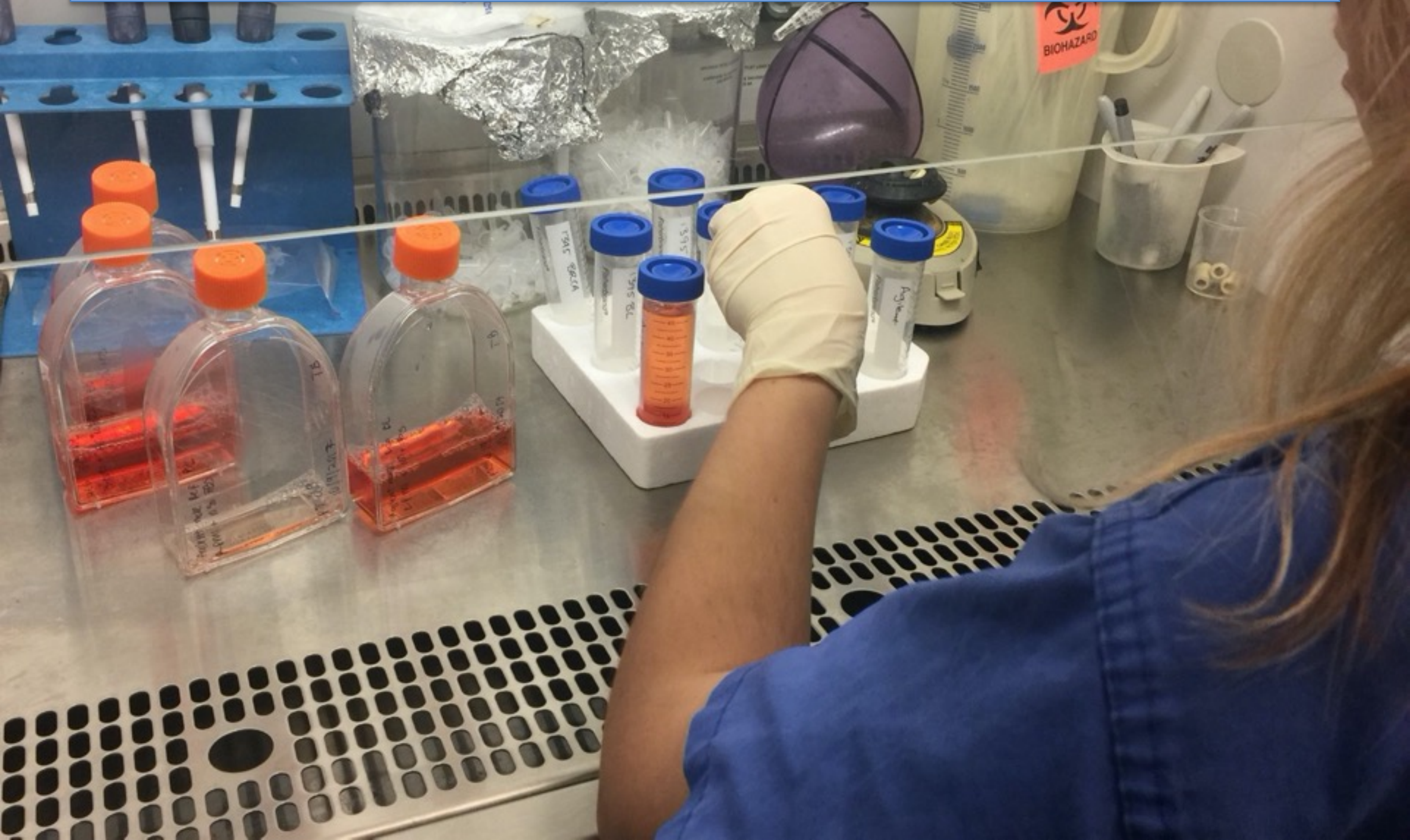

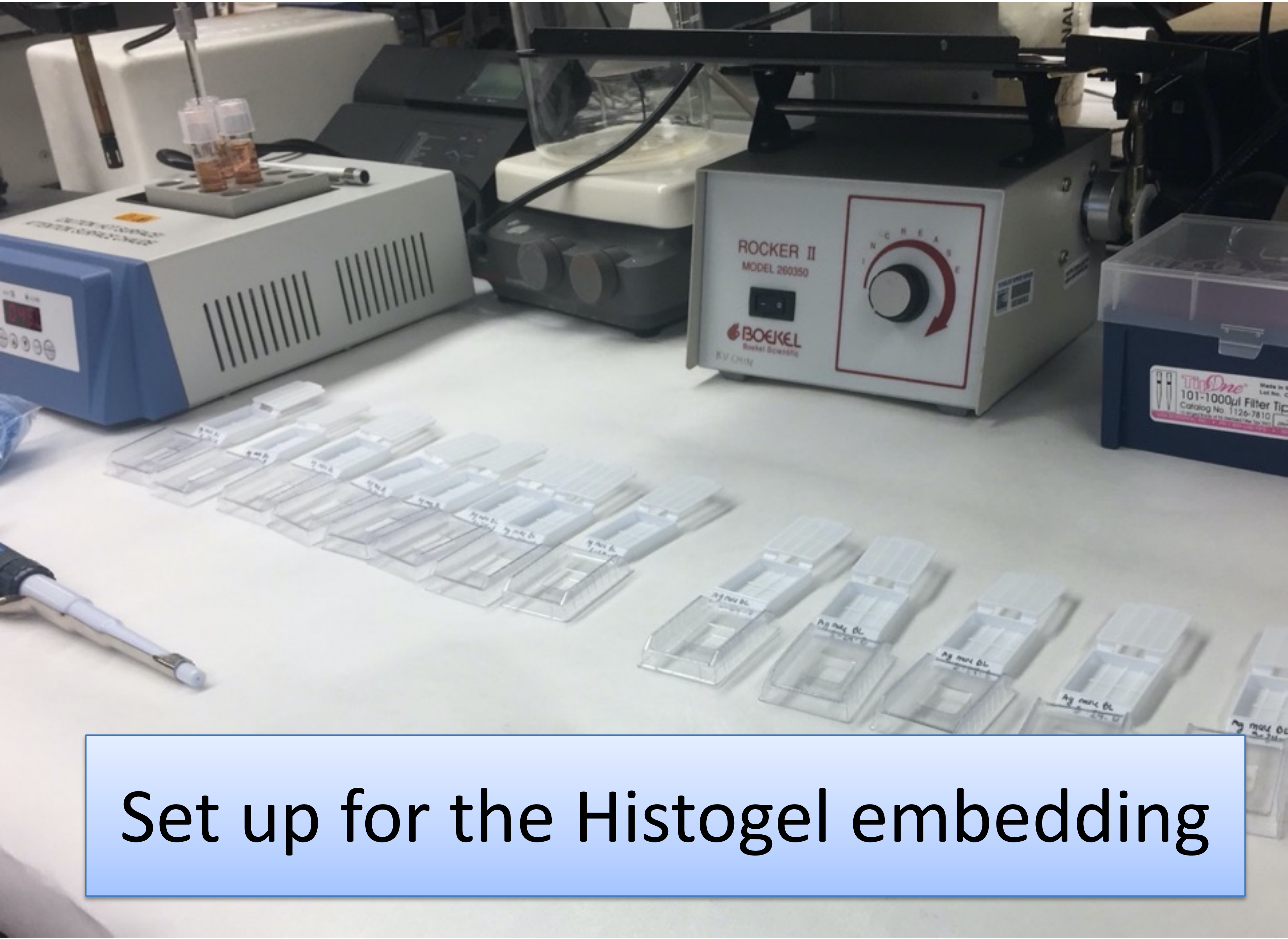

Set up for the Histogel embedding

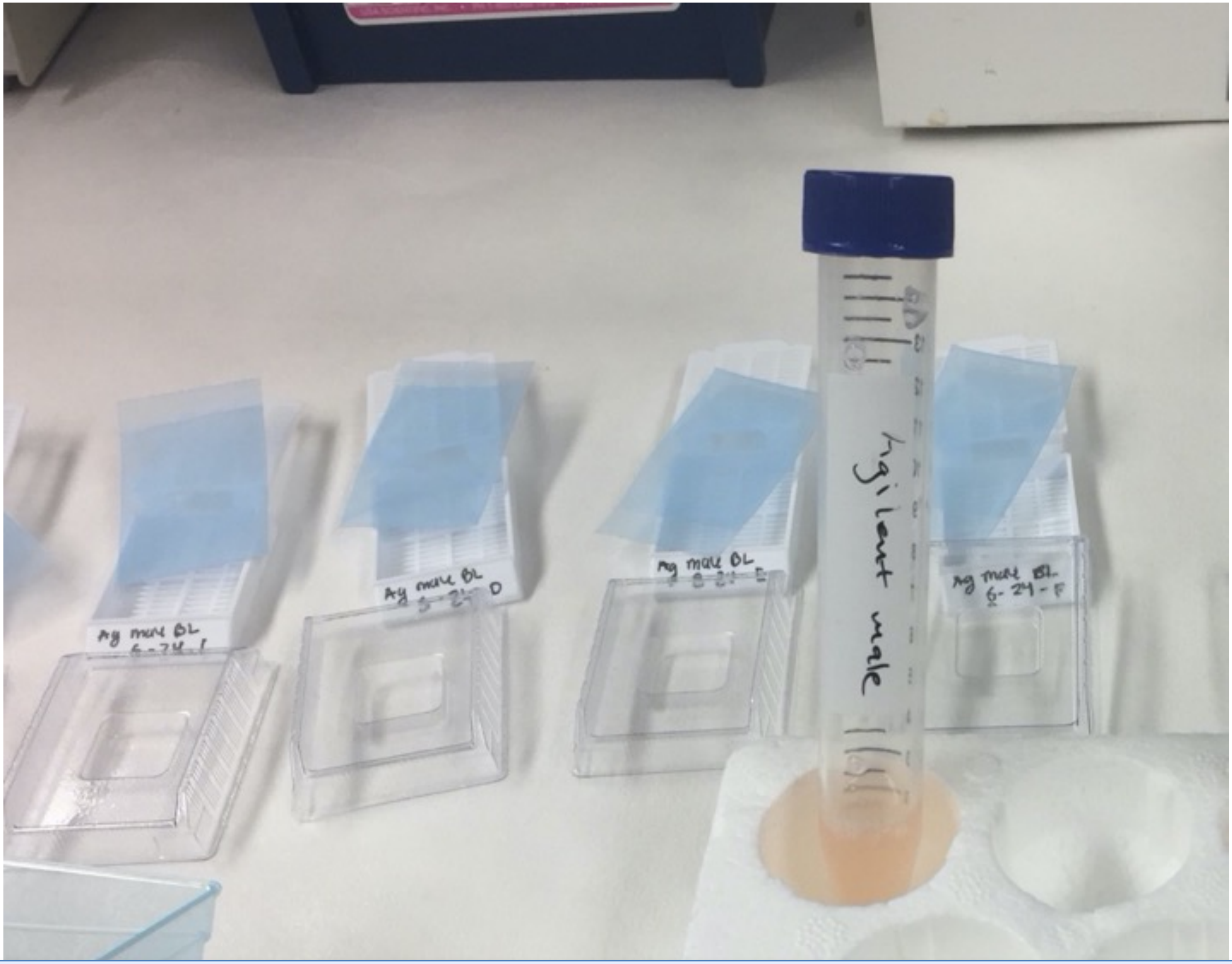

Harvested cell pellets are suspended in 10% neutral buffered formalin

In each Mold:
500uL Histogel (45°C) is added to 100uL of suspended cell line and mixed

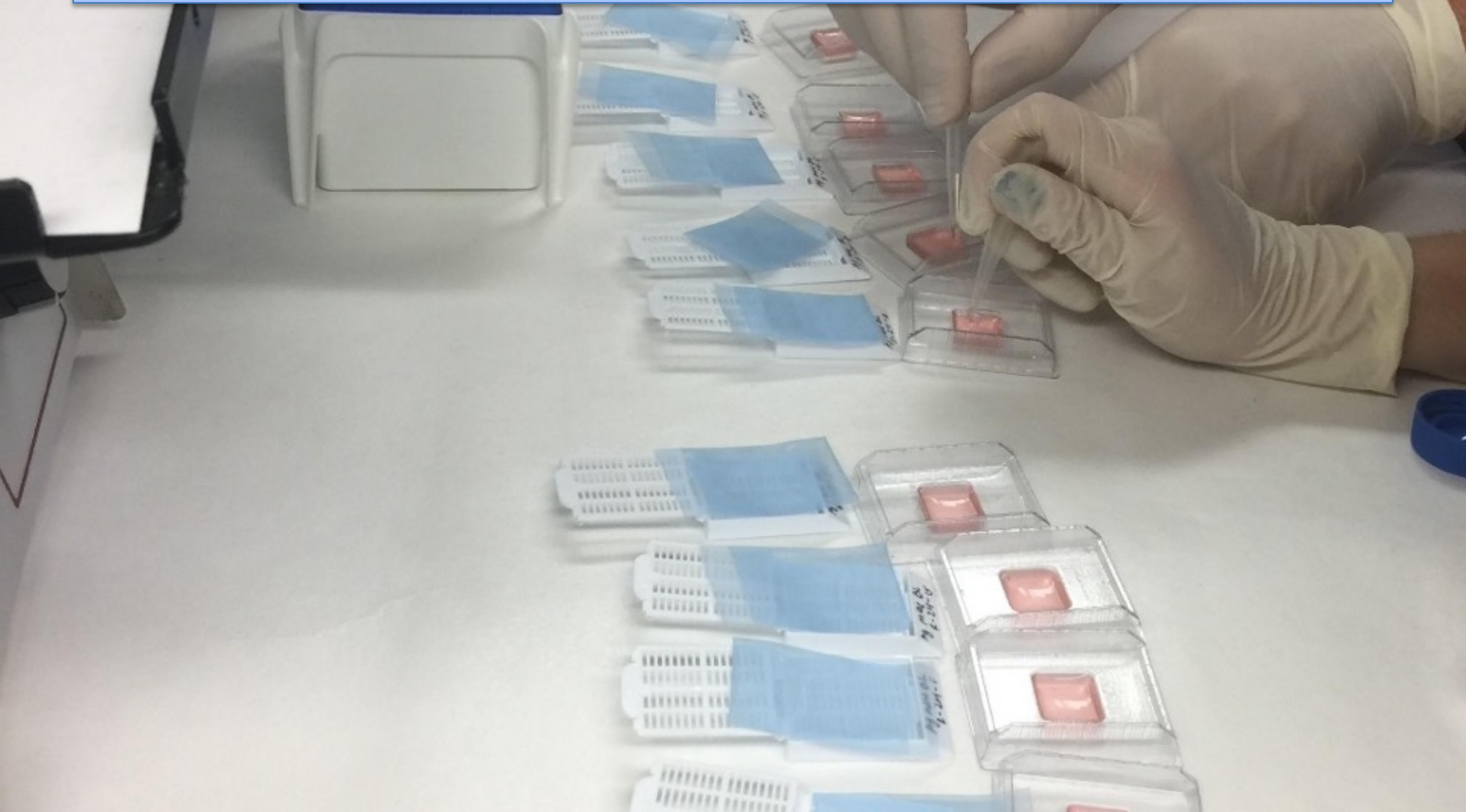

Histogel is set (~5 minutes)
Mold has about 0.5-1.0 million cells each
Cell molds are placed in Nylon Bags

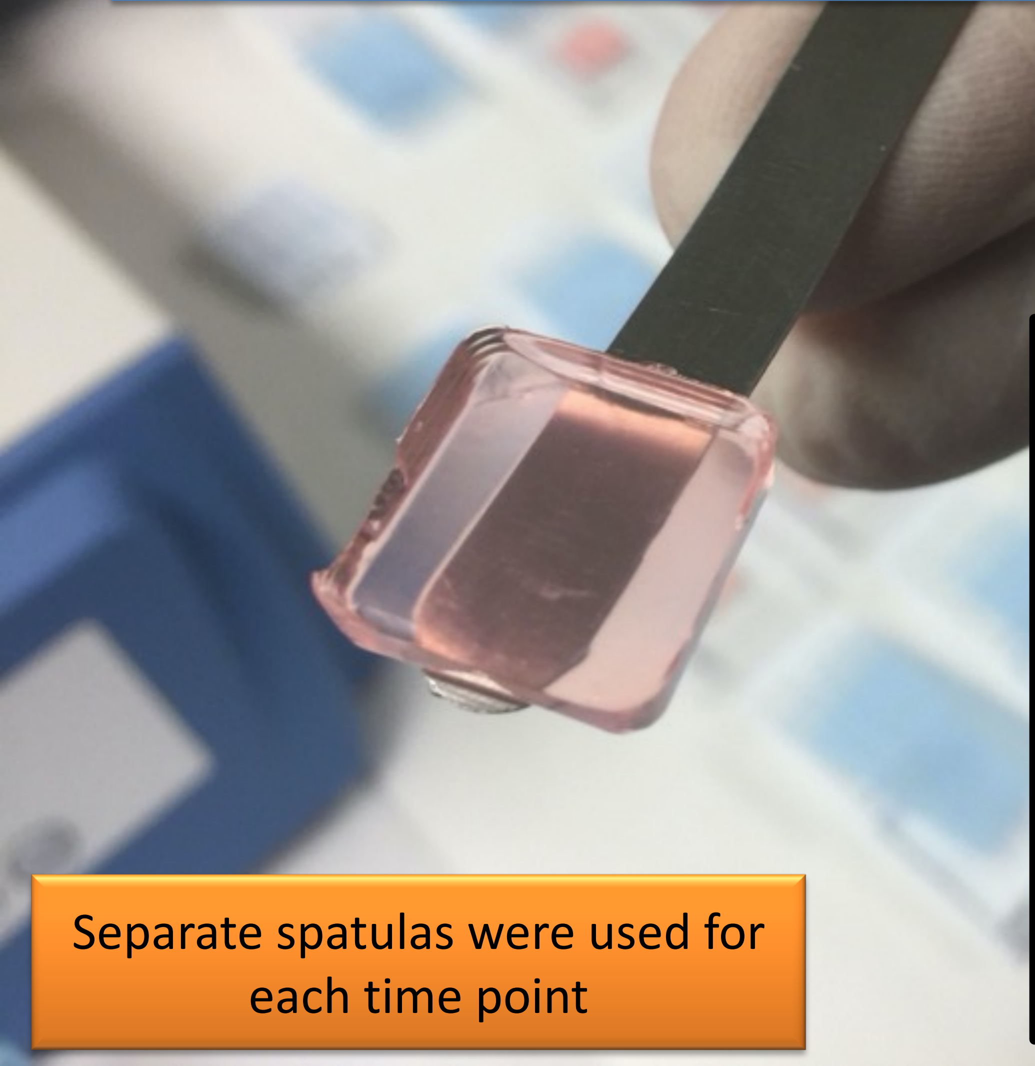

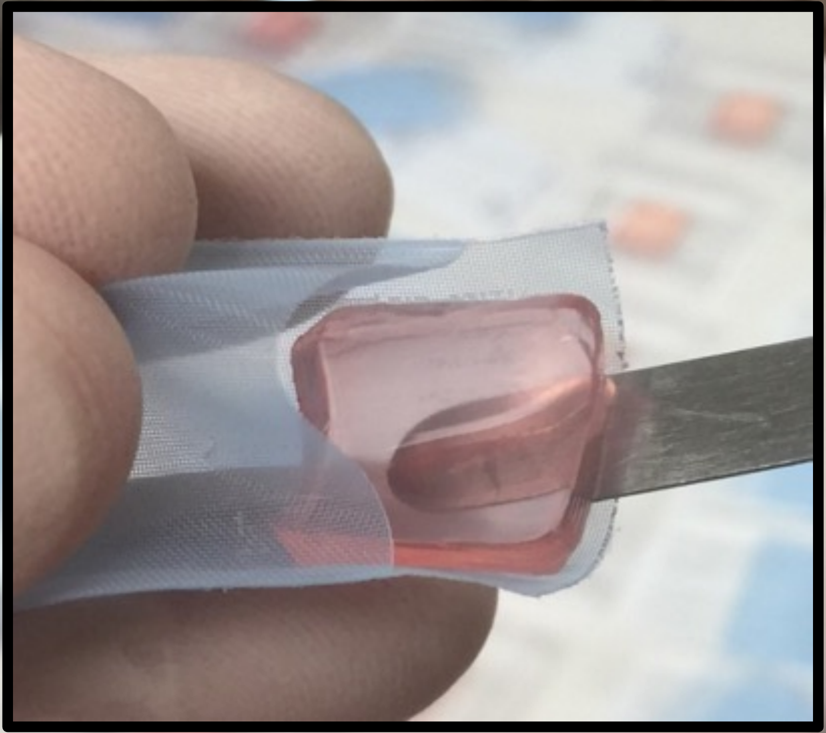

Separate spatulas were used for each time point

Nylon bags with histogel cell molds are placed in cassettes and then in 10% neutral buffered formalin for 24, 6, 2, or 1 hours.

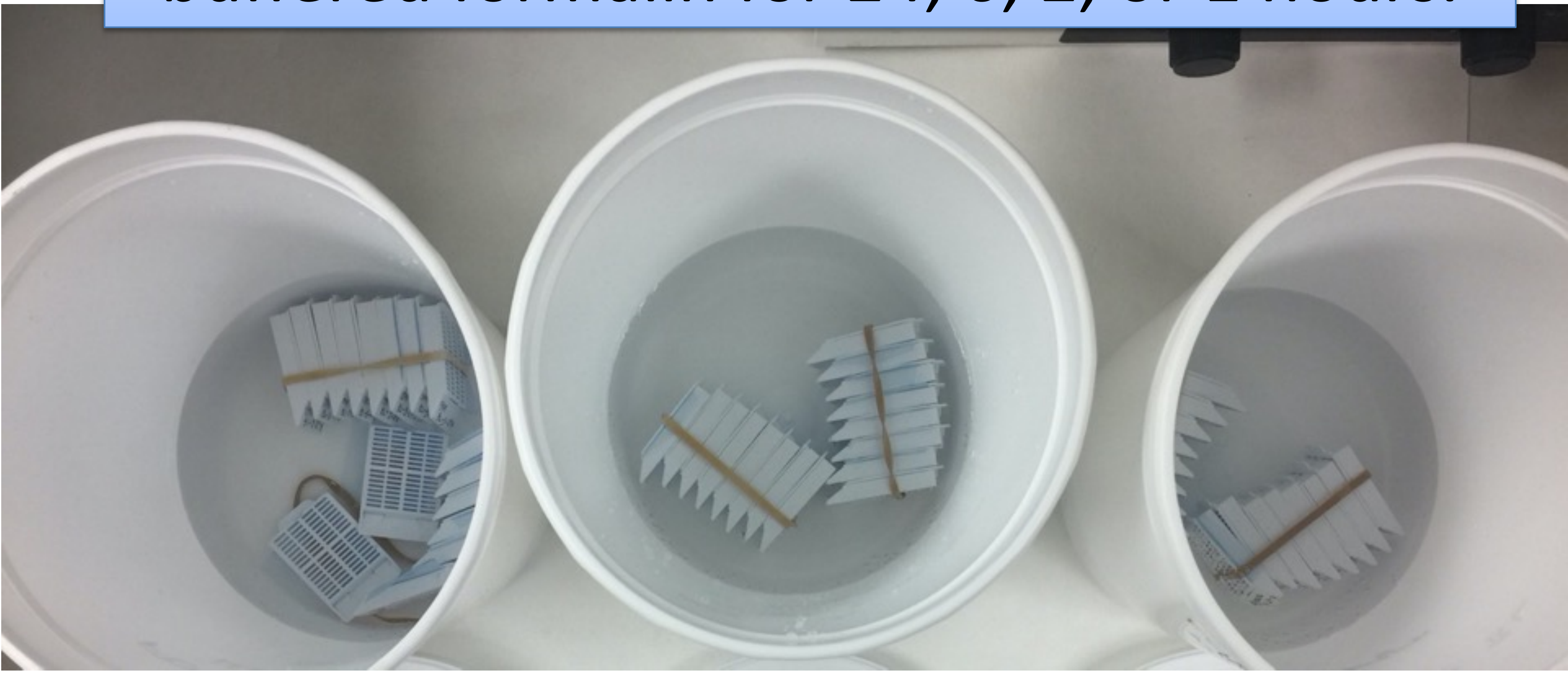

Agilent Male B-Lymphoblast Line

After 24, 6, 2 or 1 hour pre-processing formalin fixation: Cassettes are placed in tissue processor for "Routine" Run

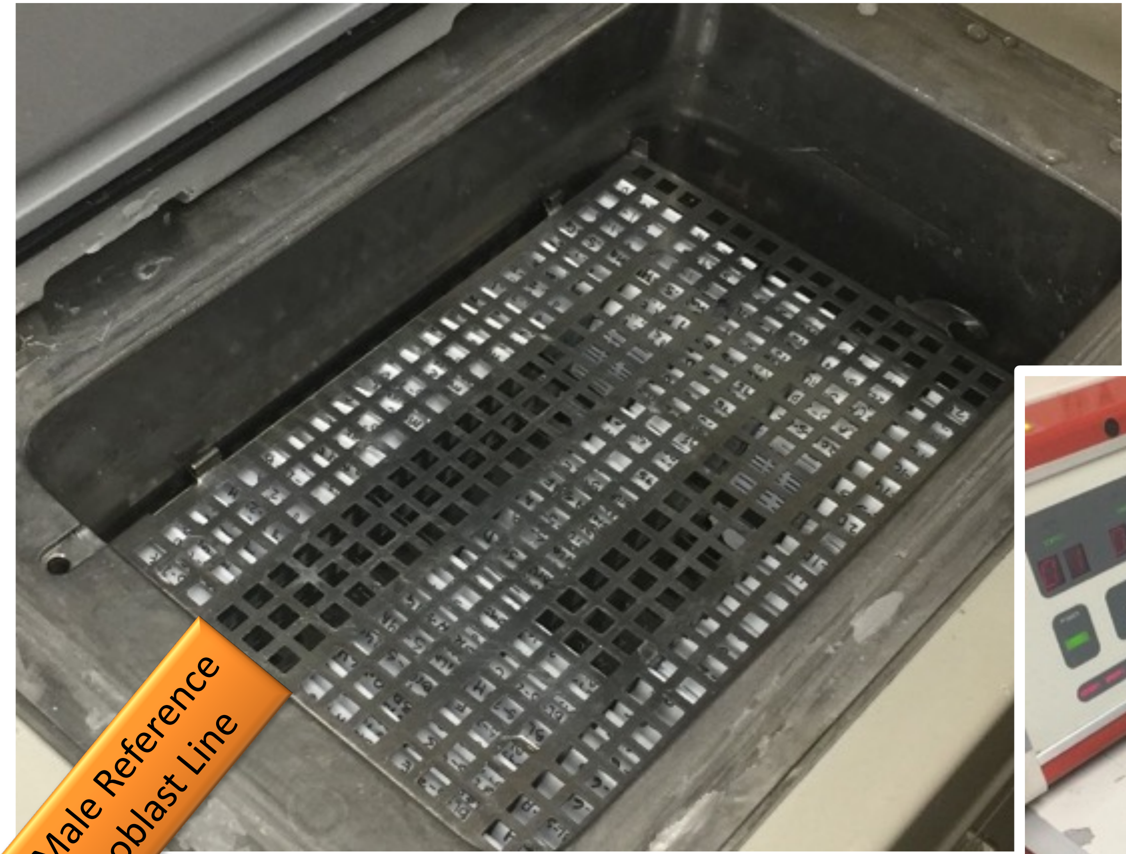

Agilent Male Reference
B-Lymphoblast Line

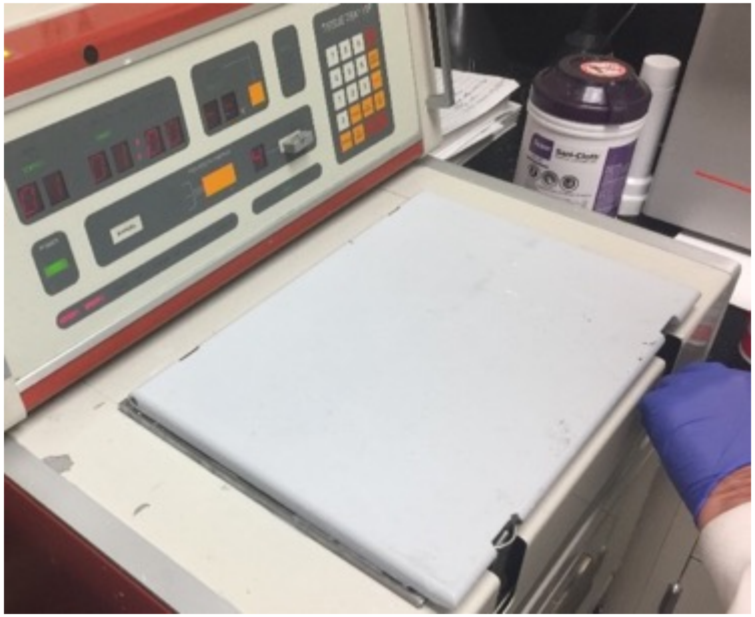

UTMC-Pathology “Routine” Run on Sakura Tissue Tek VIP 5 Tissue Processor

STATION 1	10% Neutral Buffered Formalin	1 Hour
STATION 2	10% Neutral Buffered Formalin	1 Hour
STATION 3	70% Ethanol	1 Hour
STATION 4	80% Ethanol	1 Hour
STATION 5	95% Ethanol	45 minutes
STATION 6	95% Ethanol	45 minutes
STATION 7	100% Ethanol	45 minutes
STATION 8	100% Ethanol	45 minutes
STATION 9	Xylene	45 minutes
STATION 10	Xylene	45 minutes
STATION 11	Paraffin @ 60° Celsius	30 minutes
STATION 12	Paraffin @ 60° Celsius	30 minutes
STATION 13	Paraffin @ 60° Celsius	30 minutes
STATION 14	Paraffin @ 60° Celsius	0 minutes

Cell blocks (Post-tissue processing) are embedded in paraffin

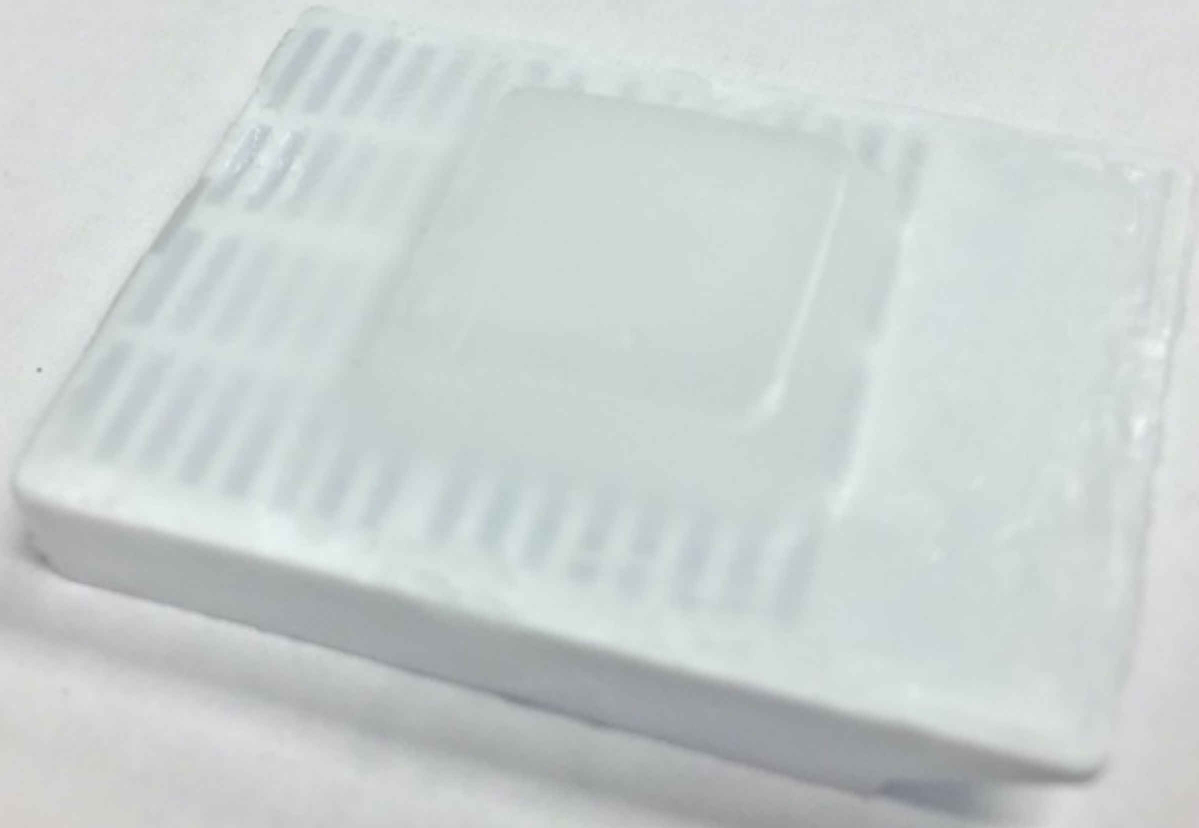

Cell blocks (Post-tissue processing) are embedded in paraffin

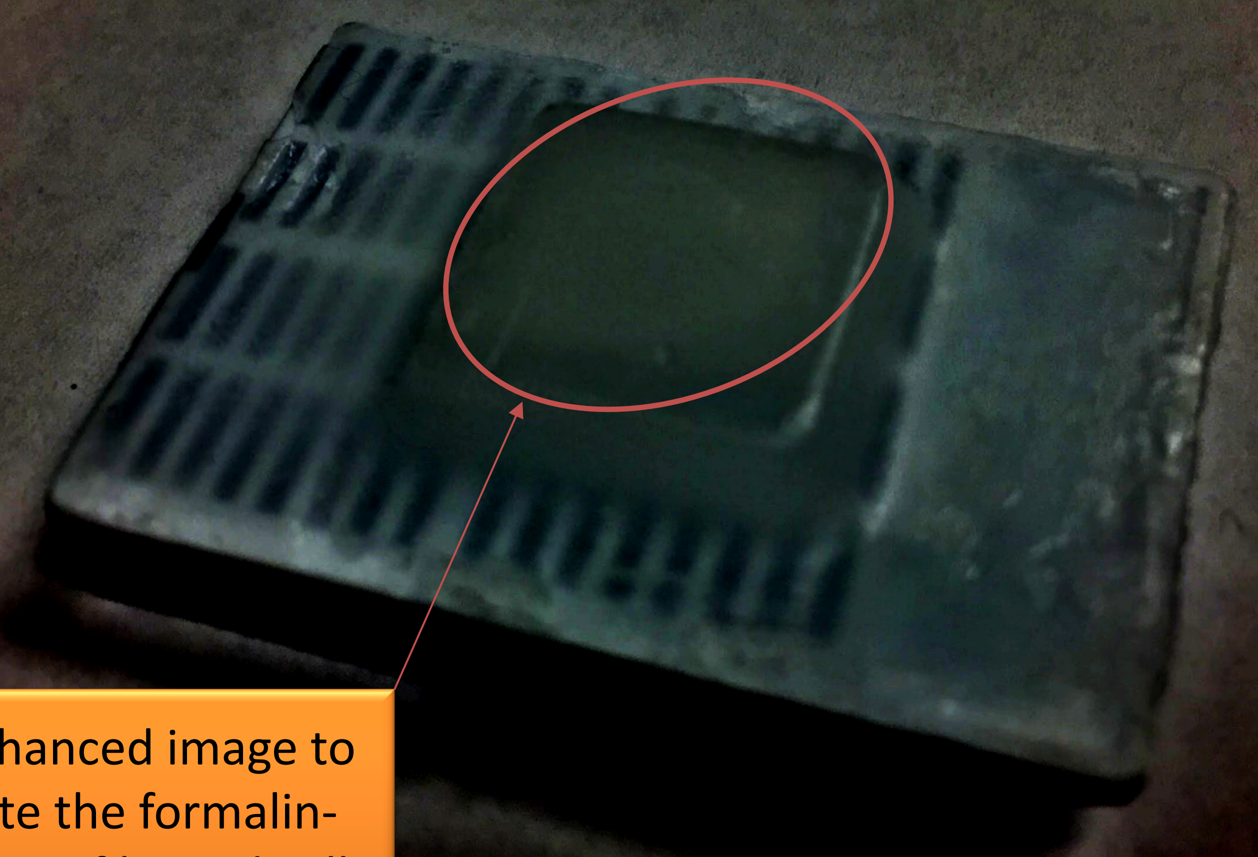

Contrast enhanced image to demonstrate the formalin-fixed paraffin infiltrated cell block embedded in paraffin

These cell blocks were then serially sectioned using a microtome at 5 micrometer thickness and this shaved materials was placed into individual Lobind Eppendorf tubes and sent to vendors for extraction, library preparation, and NGS analysis

Every 8 sections, a representative section was taken for routine microscopic analysis with H&E staining for cell-counting

Visual schematic of **Surface vs Inner** in FFPE Cell blocks

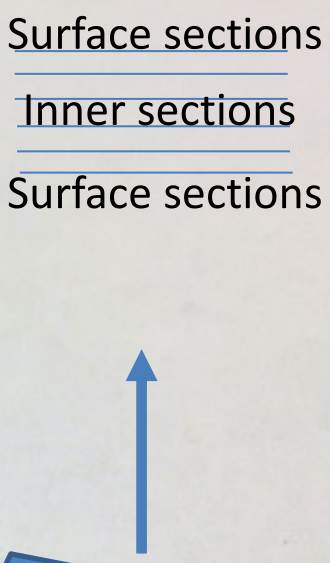

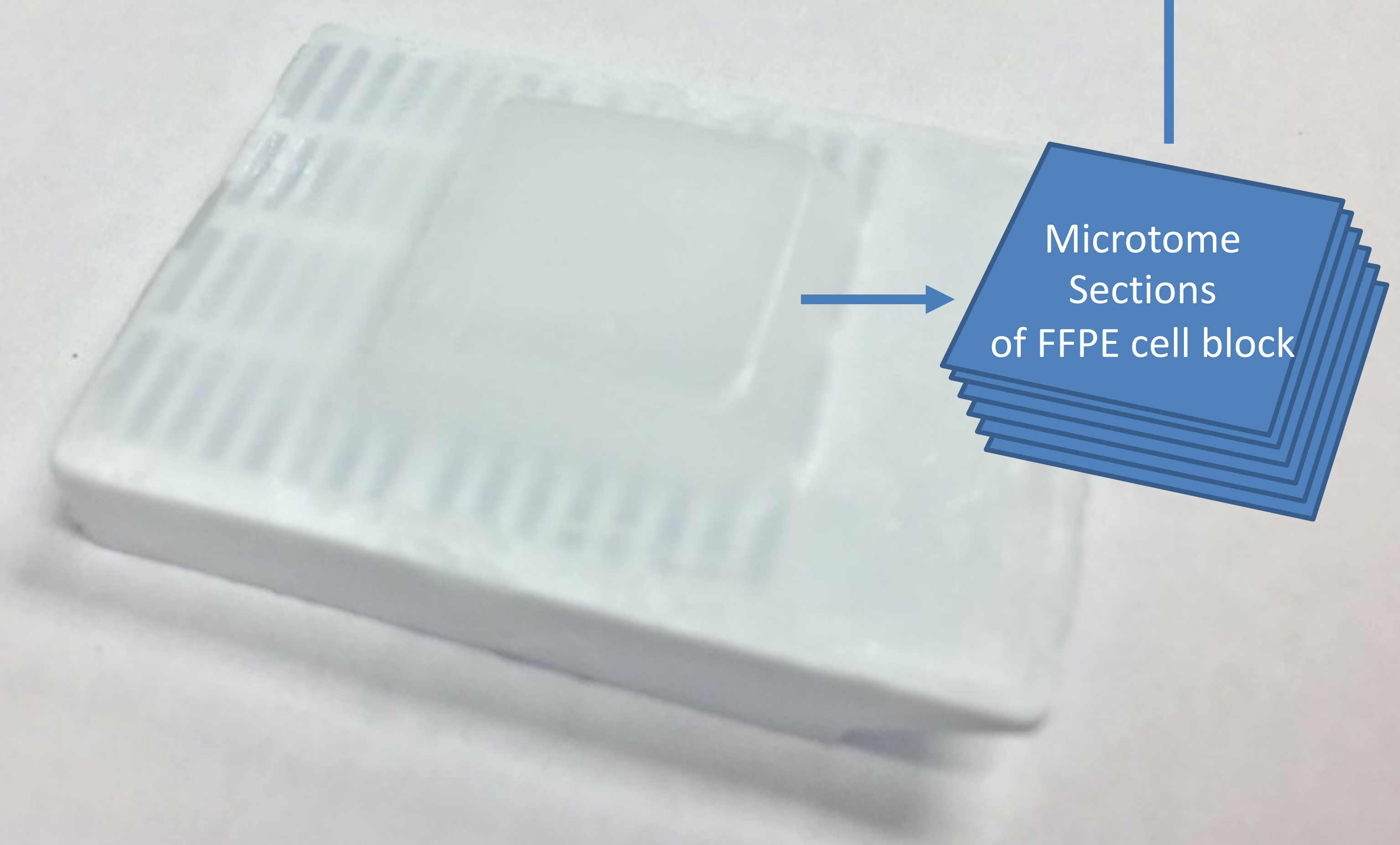

Hematoxylin and Eosin stained sections from FFPE cell blocks

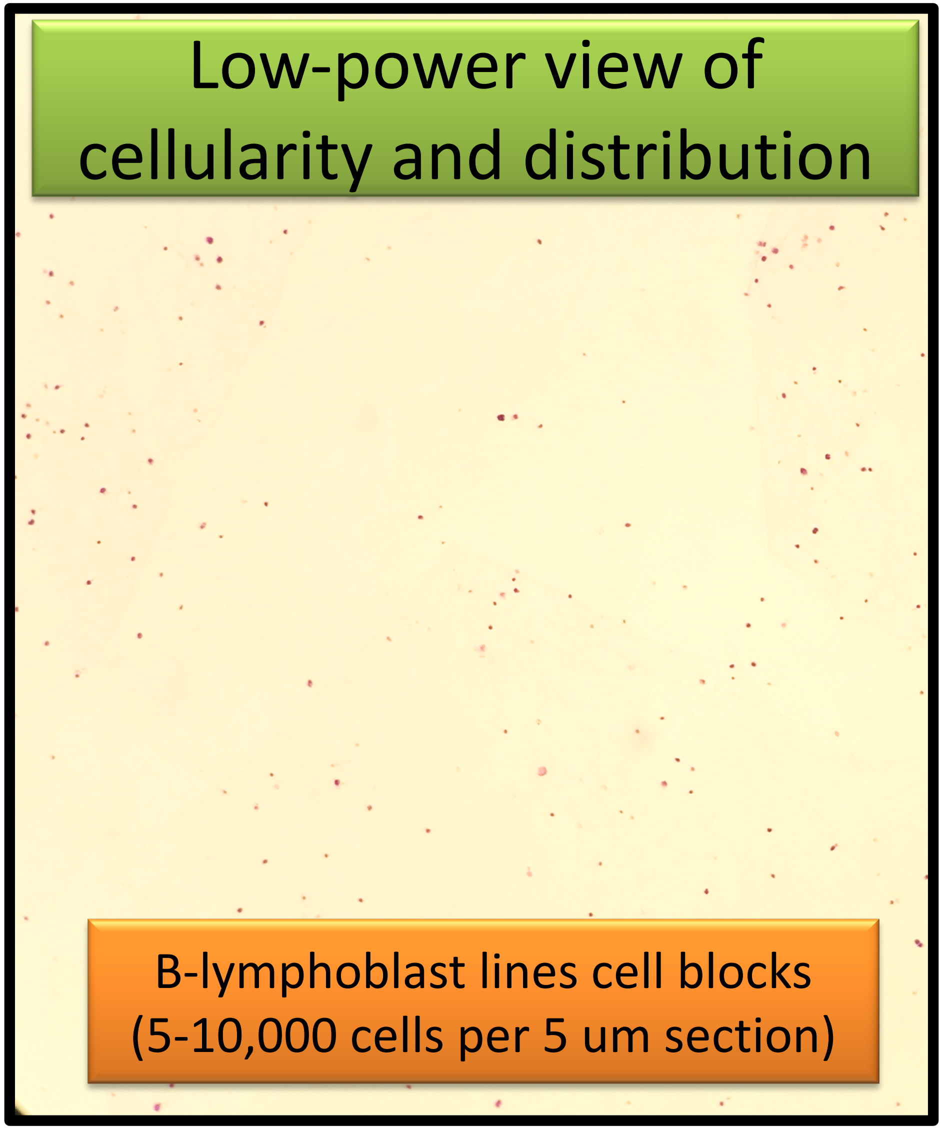

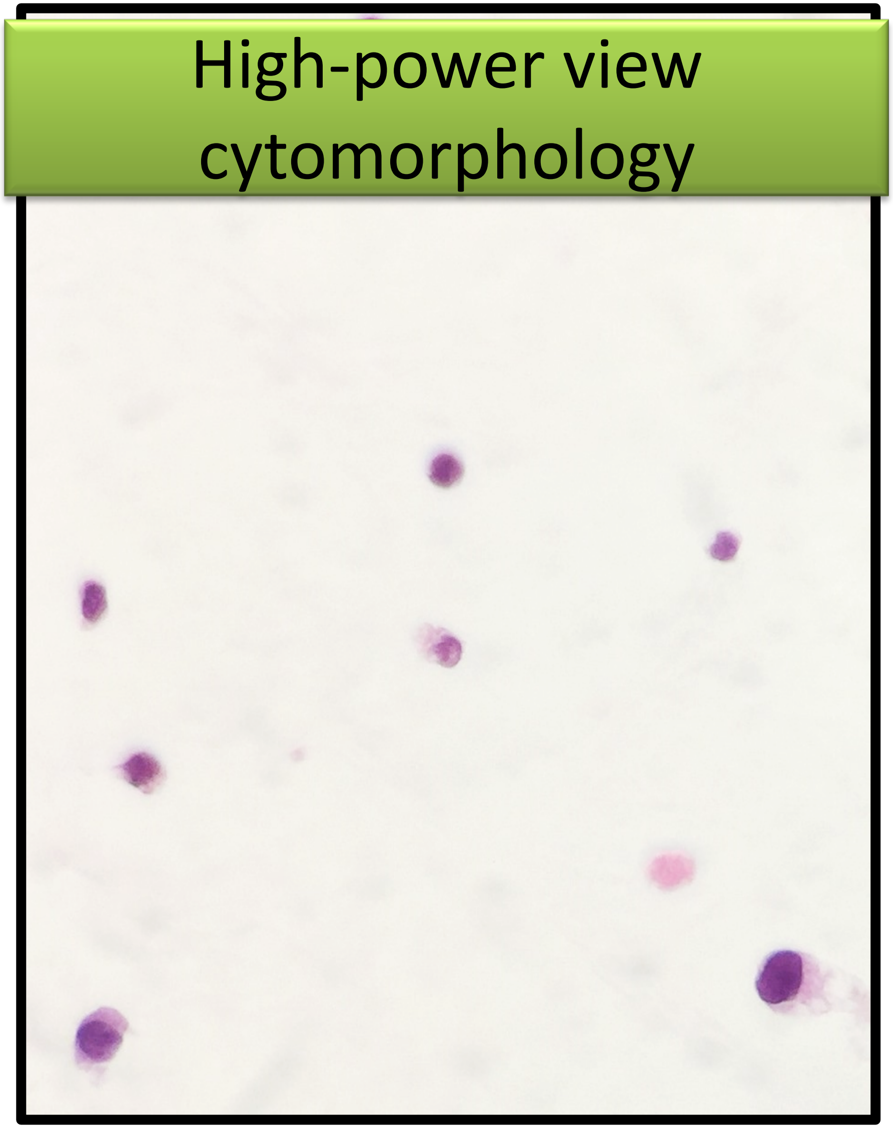

Immunohistochemical staining of sections from FFPE cell blocks

Leukocyte Common Antigen (CD45)

PanKeratin

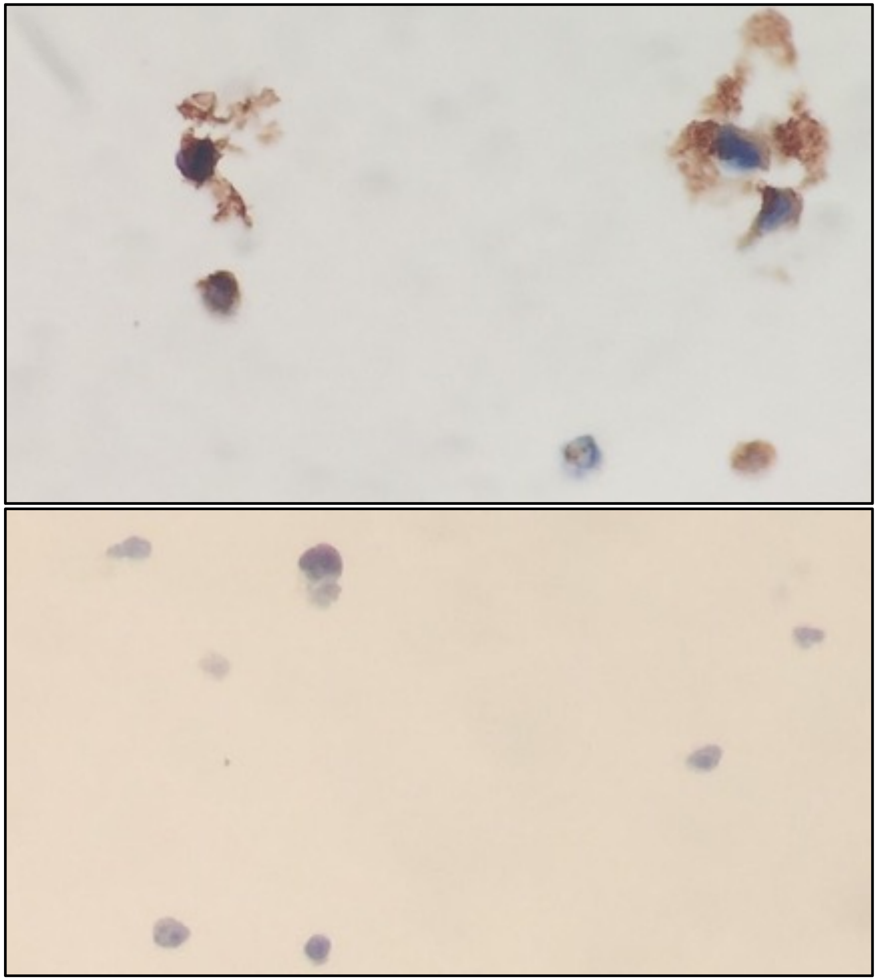

FFPE Agilent B-lymphoblast cell-line cell-block
