# Supplementary material for: Deep oncopanel sequencing reveals within block position-dependent quality degradation in FFPE processed samples
Source: Genome Biol. 2022 Jun 29;23:141. doi: 10.1186/s13059-022-02709-8 (PMC9241261; doi:10.1186/s13059-022-02709-8)
Supplement: Supplementary file 5 — Additional file 5: Table S1. List of detailed information for four participating oncology panels. [file 13059_2022_2709_MOESM5_ESM.pdf]

Table S1 | List of detailed information for four participating oncology panels

Panel code	Panel name	Genome version	Gene count	Design Region (Kbp)	Reporting Region (Kbp)
AZ650	AstraZeneca 650 genes Oncology Research Panel	hg38	650	1,808	1,808
BRP	Burning Rock DX OncoScreen Plus	hg19	523	1,631	1,072
ILM	Illumina TruSight Tumor 170	hg19	154	527	527
TFS	Thermo Fisher Oncomine Comprehensive Assay v3	hg19	146	349	289

continue…

Panel code	Reporting Region within the CTR* (Kbp)	Reporting Region outside the CTR (Kbp)	Fragmentation approach
AZ650	1116	692	Enzymatic fragmentation
BRP	823	250	Covaris M220/Focused-Ultrasonicators with AFA Technology
ILM	349	178	Covaris / Focused-Ultrasonicators
TFS	174	115	No fragmentation as PCR based target amplification

continue…

Panel code	Enrichment	Sequencing platform	Read length	UMI	Avg. read count
AZ650	capture based	HiSeq 4000 or NovaSeq 6000	2 x 150bp	Yes	40.8M (consensus reads)
BRP	capture based	NovaSeq 6000	2 x 150bp	No	74.3M
ILM	capture based	NextSeq 550	2 x 101bp	No	80.7M
TFS	amplicon based	IonTorrent S5 XL	113 bp (average)	No	15.4M (mapped)

continue…

Panel code	Read mapping tool	Variant caller	VAF threshold
AZ650	bwa mem v0.7.17	fabio v1.0.0 and VarDict v1.7.0	2%
BRP	BWA aligner 0.7.10	VarScan v2.4.3	1%
ILM	iSAAC aligner	Pisces variant caller	2.6%
TFS	TMAP	Torrent Variant Caller (TVC)	2.5%

* CTR is the consensus high confidence targeted region (see Methods for details).
